# Risk of bias of prognostic models developed using machine learning: a systematic review in oncology

**DOI:** 10.1186/s41512-022-00126-w

**Published:** 2022-07-07

**Authors:** Paula Dhiman, Jie Ma, Constanza L. Andaur Navarro, Benjamin Speich, Garrett Bullock, Johanna A. A. Damen, Lotty Hooft, Shona Kirtley, Richard D. Riley, Ben Van Calster, Karel G. M. Moons, Gary S. Collins

**Affiliations:** 1 Centre for Statistics in Medicine, Nuffield Department of Orthopaedics, Rheumatology and Musculoskeletal Sciences, University of Oxford, Oxford, OX3 7LD UK; 2 NIHR Oxford Biomedical Research Centre, Oxford University Hospitals NHS Foundation Trust, Oxford, UK; 3 Julius Center for Health Sciences and Primary Care, University Medical Center Utrecht, Utrecht University, Utrecht, The Netherlands; 4 Cochrane Netherlands, University Medical Center Utrecht, Utrecht University, Utrecht, The Netherlands; 5 Department of Clinical Research, Basel Institute for Clinical Epidemiology and Biostatistics, University Hospital Basel, University of Basel, Basel, Switzerland; 6 Nuffield Department of Orthopaedics, Rheumatology, and Musculoskeletal Sciences, University of Oxford, Oxford, UK; 7 Centre for Prognosis Research, School of Medicine, Keele University, Staffordshire, ST5 5BG UK; 8 Department of Development and Regeneration, KU Leuven, Louvain, Belgium; 9 Department of Biomedical Data Sciences, Leiden University Medical Center, Leiden, the Netherlands; 10 EPI-Centre, KU Leuven, Louvain, Belgium

**Keywords:** Prediction modelling, Machine learning, Systematic review, Risk of bias

## Abstract

**Background:**

Prognostic models are used widely in the oncology domain to guide medical decision-making. Little is known about the risk of bias of prognostic models developed using machine learning and the barriers to their clinical uptake in the oncology domain.

**Methods:**

We conducted a systematic review and searched MEDLINE and EMBASE databases for oncology-related studies developing a prognostic model using machine learning methods published between 01/01/2019 and 05/09/2019. The primary outcome was risk of bias, judged using the Prediction model Risk Of Bias ASsessment Tool (PROBAST). We described risk of bias overall and for each domain, by development and validation analyses separately.

**Results:**

We included 62 publications (48 development-only; 14 development with validation). 152 models were developed across all publications and 37 models were validated. 84% (95% CI: 77 to 89) of developed models and 51% (95% CI: 35 to 67) of validated models were at overall high risk of bias. Bias introduced in the analysis was the largest contributor to the overall risk of bias judgement for model development and validation. 123 (81%, 95% CI: 73.8 to 86.4) developed models and 19 (51%, 95% CI: 35.1 to 67.3) validated models were at high risk of bias due to their analysis, mostly due to shortcomings in the analysis including insufficient sample size and split-sample internal validation.

**Conclusions:**

The quality of machine learning based prognostic models in the oncology domain is poor and most models have a high risk of bias, contraindicating their use in clinical practice. Adherence to better standards is urgently needed, with a focus on sample size estimation and analysis methods, to improve the quality of these models.

**Supplementary Information:**

The online version contains supplementary material available at 10.1186/s41512-022-00126-w.

## Background

Clinical prediction models use multiple variables (predictors) in combination (such as demographics, clinical information, test results and biomarker values), to estimate the risk of existing (diagnostic) or future (prognostic) patient health outcomes. Many medical decisions across all specialties are informed by prediction models [1–7]. In particular, in oncology prediction models are used to assess an individual’s risk of developing cancer, help cancer diagnosis, assess cancer prognosis and guide treatment [8–13]. However, compared to the number of prediction models that are developed in oncology, very few are used in daily practice and many models contribute to research waste [14–17]. This problem might be further exacerbated with the rapidly growing use of machine learning methods, which refers to a broad set of computational (artificial intelligence, AI) approaches including neural networks and random forests.

Machine learning is often portrayed as offering many advantages (over traditional statistical modelling) for developing prediction models, including offering more flexible modelling, ability to analyse ‘big’, non-linear, and high dimensional data, and modelling complex clinical scenarios. However, these advantages have not yet materialised into patient benefit as most models are not yet being used in clinical practice [18–23]. Given the increasing concern about the methodological quality and risk of bias of prediction model studies, caution is warranted and the lack of uptake of models in medical practice is not surprising [18, 21, 22, 24].

Though there is much evidence about the risk of bias of prediction models developed using traditional statistical modelling methods, there is a dearth of research evaluating machine learning methods. The aim of this study was to evaluate the risk of bias of prognostic prediction models developed using machine learning (as defined by the authors of the primary studies) in the field of Oncology.

## Methods

We conducted a review of prognostic (future outcome) risk prediction modelling studies within the oncology domain that use machine learning methods (as defined by the authors of the primary studies).

### Protocol registration and reporting standards

This study was registered with PROSPERO (ID: CRD42019140361) and reported using the Preferred Reporting Items for Systematic Reviews and Meta-Analyses (PRISMA) guideline [25, 26].

### Information sources

We searched the MEDLINE (via OVID) and Embase (via OVID) medical literature databases for prognostic prediction modelling studies developed using machine learning methods within the oncology field and published between 1 Jan 2019 to 5 September 2019 (the date that the search was conducted).

The full search strategies for both databases are provided in Supplementary tables 1 and 2. The search terms included relevant Mesh and EMTREE headings and free-text terms. We searched in the title, abstract or keyword fields, for general modelling terms (such as “machine learning” and “deep learning”), more specific machine learning modelling terms (such as “random forest”, “support vector machine” and “neural networks”), cancer terms (such as “cancer”, “malignant” and “carcinoma”), prediction-related search terms (such as “prediction”, “prognostic” and “risk of”) and specific model performance terms (such as “discrimination” and “calibration”). Modelling, cancer, and prediction terms were combined with ‘and’ to retrieve publications meeting all three sets of search criteria. The search was limited to retrieve studies published in 2019 only to ensure that a contemporary sample of studies were assessed in the review. Apart from the date range specified no other limits were applied to the search. An information specialist (SK) was involved in the development of the search strategy for both databases.

### Eligibility criteria

Published studies developing a prognostic model using machine learning methods, as defined by authors of the primary report, within the oncology field in 2019 were included.

The boundary between machine learning and statistical (regression-based) methods for prediction is unclear and often cultural rather than based on specific methods [27]. Whilst some methods, such as neural networks typically fall into machine learning taxonomy, other methods such as logistic regression are frequently ascribed to both domains. We therefore included studies developing a prognostic model if the modelling method was defined as machine learning by the authors of the primary report. For example, studies using logistic regression were included if they were explicitly described as machine learning by the primary study authors anywhere in the primary report, else it was excluded. Publications also had to fulfil the following inclusion and exclusion criteria:

#### Inclusion Criteria

- Development of a prediction model for individualised prognosis using machine learning methods, as defined by authors of the primary report:
  1. in the oncology domain
  2. for any health-related outcome
  3. for any outcome measurement (e.g., continuous, binary, ordinal, multinomial, time-to-event)
  4. using at least two or more predictors (prognostic factors)
  5. using any study design
    experimental studies (including randomised trials)
    observational studies (including prospective studies, retrospective studies, cohort studies, case-control studies)

- English language studies

#### Exclusion Criteria

- Studies that only evaluated the performance of an existing prediction model (e.g., an external validation study)
- Imaging studies, or studies using imaging parameters as candidate predictors in the model
- Speech recognition/voice pattern studies, or studies using speech parameters as candidate predictors
- Lab-based studies
  - Genetic studies, or studies using genetic risk factors as candidate predictors
  - Molecular studies, or studies using molecular markers as candidate predictors

- Risk or prognostic factor studies, primarily interested in the association of risk factors with the outcome
- Secondary research (e.g., reviews of prediction models)
- Conference abstracts

### Study selection, data extraction and data management

Publications from MEDLINE and Embase were imported into Endnote reference software where they were de-duplicated and then imported into Rayyan web application where they were screened [28, 29].

Two independent researchers (PD, JM) screened the titles and abstracts of the identified publications. Two independent researchers, from a combination of five reviewers (PD, JM, GB, BS, CLAN) reviewed the full text for potentially eligible publications and performed a double data extraction of eligible publications. One researcher screened all publications (PD) and four researchers collectively screened the same publications (JM, GB, BS, CLAN). Disagreements were discussed and adjudicated by a third reviewer (GSC), where necessary.

The primary outcome was risk of bias for each developed model and was assessed using the Prediction model Risk Of Bias ASsessment Tool (PROBAST) [30, 31]. PROBAST examines the extent to which a model’s risk predictions are likely to be accurate when applied in new individuals and depends on four domains (Table 1): participants, predictors, outcomes and analysis. The data extraction form was developed using PROBAST, in combination with the CHARMS checklist [32], and applied to each model based on information within the model development article (including any external validation if reported within the development article). The extraction form was piloted among all the five reviewers using five eligible publications. Results of the pilot were discussed, and data extraction items were clarified amongst all reviewers. Text for items in the PROBAST assessment were not changed, instead notes were added to each item for clarification and to ensure consistent data extraction. The data extraction form was implemented using Research Data Capture (REDCap) software [33].

**Table 1:** Study characteristics of the 62 included publications, by study type

	**All (*n* = 62)**	**Development only (*n* = 48)**	**Development and external validation (*n* = 14)**
	***n* (%)**	***n* (%)**	***n* (%)**
**Study characteristics**			
**Cancer type**			
Lung	8 (12.9)	6 (12.5)	2 (14.3)
Breast	6 (9.7)	6 (12.5)	-
Colon/colorectal/rectal	6 (9.7)	3 (6.3)	3 (21.4)
Pancreatic	3 (4.8)	1 (2.1)	2 (14.3)
Liver	2 (3.2)	2 (4.2)	-
Gastric	3 (4.7)	3 (6.3)	-
Head and neck	5 (8.1)	5 (10.4)	-
Spinal	4 (6.5)	4 (8.3)	-
Brain (inc. meningioma, glioblastoma)	5 (8.1)	4 (8.3)	1 (7.1)
Oral (inc. nasopharyngeal carcinoma)	3 (4.8)	2 (4.2)	1 (7.1)
Gynaecological (inc. cervical, ovarian, endometrial)	6 (9.7)	5 (10.4)	1 (7.1)
Prostate/penile	5 (8.1)	4 (8.3)	1 (7.1)
Skin (inc. melanoma)	2 (3.2)	1 (2.1)	1 (7.1)
Other*	4 (6.5)	2 (4.2)	2 (14.3)
**Target population**			
Cancer patients	55 (88.7)	43 (89.6)	12 (85.7)
General population	6 (9.7)	4 (8.3)	2 (14.3)
Unclear	1 (1.6)	1 (2.1)	-
**Outcome**			
Binary	48 (77.4)	40 (83.3)	8 (57.1)
* Complication*	11	11	-
* Survival*	8	7	1
* Recurrence*	7	4	3
* Cancer occurrence*	6	4	2
* Metastases*	4	3	1
* Treatment response*	4	4	-
* Mortality*	3	2	1
* Resection*	3	3	-
* Screening*	1	1	-
* Progression*	1	1	-
Continuous	1 (1.6)	-	1 (7.1)
* Length of stay*	1	-	1
Multinomial	2 (3.2)	2 (4.2)	-
* Test result*	1	1	-
* Treatment response*	1	1	-
Time to event	11 (17.7)	6 (12.5)	5 (35.7)
* Overall survival*	7	3	4
* Cancer specific survival*	1	-	1
* Cause specific mortality*	1	1	-
* Disease specific survival*	1	1	-
* Progression free survival*	1	1	-

^*^Other includes peritoneal carcinomatosis, incurable cancer (various), leukaemia, malignant peripheral nerve sheath tumour

### Data items

Descriptive information was extracted on the overall publication, including cancer type, study type, data source/study design, target population, type of prediction outcome, number and type of machine learning models used, setting, intended use and aim of the clinical prediction model. Items were extracted separately for the development and, if done, for validation of the models.

Items for PROBAST assessment consists of 20 signalling questions across the four domains for which details were extracted and further criteria of how the signalling questions were used to inform the risk of bias assessment can be found in Supplementary tables 3 and 4.

### Summary measures and synthesis of results

Findings were summarised using descriptive statistics with logit-transformed 95% confidence intervals and visual plots, alongside a narrative synthesis. Analysis and synthesis of data was presented overall and by study type (i.e., studies only describing the development of a model and studies describing both the development and validation of a model).

Risk of bias was assessed separately for the development and validation of each model. We assessed the risk of bias for the reported primary outcome. If the primary outcome was not explicitly specified, we took the first reported outcome in the results to be the primary outcome. The risk of bias was assessed for each model using the study level information. If more than one model was developed and validated for the primary outcome, we assumed the risk of bias profile for the ‘participants’, ‘predictors’ and ‘outcomes’ domains were the same for each model, unless additional information was reported for any specific model type. For the ‘analysis’ domain, we similarly assumed the same risk of bias profile across all models within a study if more than one model was developed and validated for signalling questions 4.1., 4.3. and 4.4.-4.9. For signalling question 4.2. ‘Were continuous and categorical predictors handled appropriately?’, we assumed these to be ‘Yes/Probably yes’ for flexible and ensemble machine learning models, unless other methods to handle continuous predictors were explicitly stated, as flexible and ensemble machine learning methods would implicitly handle continuous predictors non-linearly.

Signalling questions for each risk of bias domain were scored with ‘Yes/Probably Yes’ (‘Y/PY’), ‘No/Probably No’ (‘N/PN’) and ‘No information’. These scores were then combined to judge the risk of bias introduced by each domain. Analysis signalling questions 4.5. ‘*Was selection of predictors based on univariable analysis avoided?*’, 4.8. ‘*Were model overfitting and optimism in model performance accounted for?*’ and 4.9. ‘*Do predictors and their assigned weights in the final model correspond to the results from the reported multivariable analysis?*’ were not applicable to validation analyses. We calculated the number of signalling questions linked to a high risk of bias (answered ‘No/Probably No’ (‘N/PN’)) for each study and report the median number across all development and validation analyses.

All domains were considered ‘Low’ and ‘High’ risk of bias if all signalling questions were answered ‘Y/PY’ and ‘N/PN’, respectively. If one or more of the signalling questions were answered ‘N/PN’, models could still be considered as ‘Low’ risk of bias and this was evaluated per study. ‘Unclear’ risk of bias was assigned if insufficient information was available for signalling questions and if no other question put the domain at high risk of bias.

The risk of bias introduced by each domain was used to judge the overall risk of bias for the development and, if done, the validation of each model. Overall low risk of bias was judged if all domains were considered low risk of bias, overall high risk of bias was judged if at least 1 domain was considered high risk of bias, and overall unclear risk of bias was judged if at least one domain was considered unclear risk of bias and all other domains were low risk of bias. Each model was judged as ‘Low’, ‘High’ and ‘Unclear’ risk of bias, which was compared between development-only and development with validation studies. We calculated the number of domains at high risk of bias for each study and report the median number across all development and validation analyses.

Applicability of included studies was not evaluated. All analyses were carried out in Stata v15 [34].

## Results

**Fig. 1:**
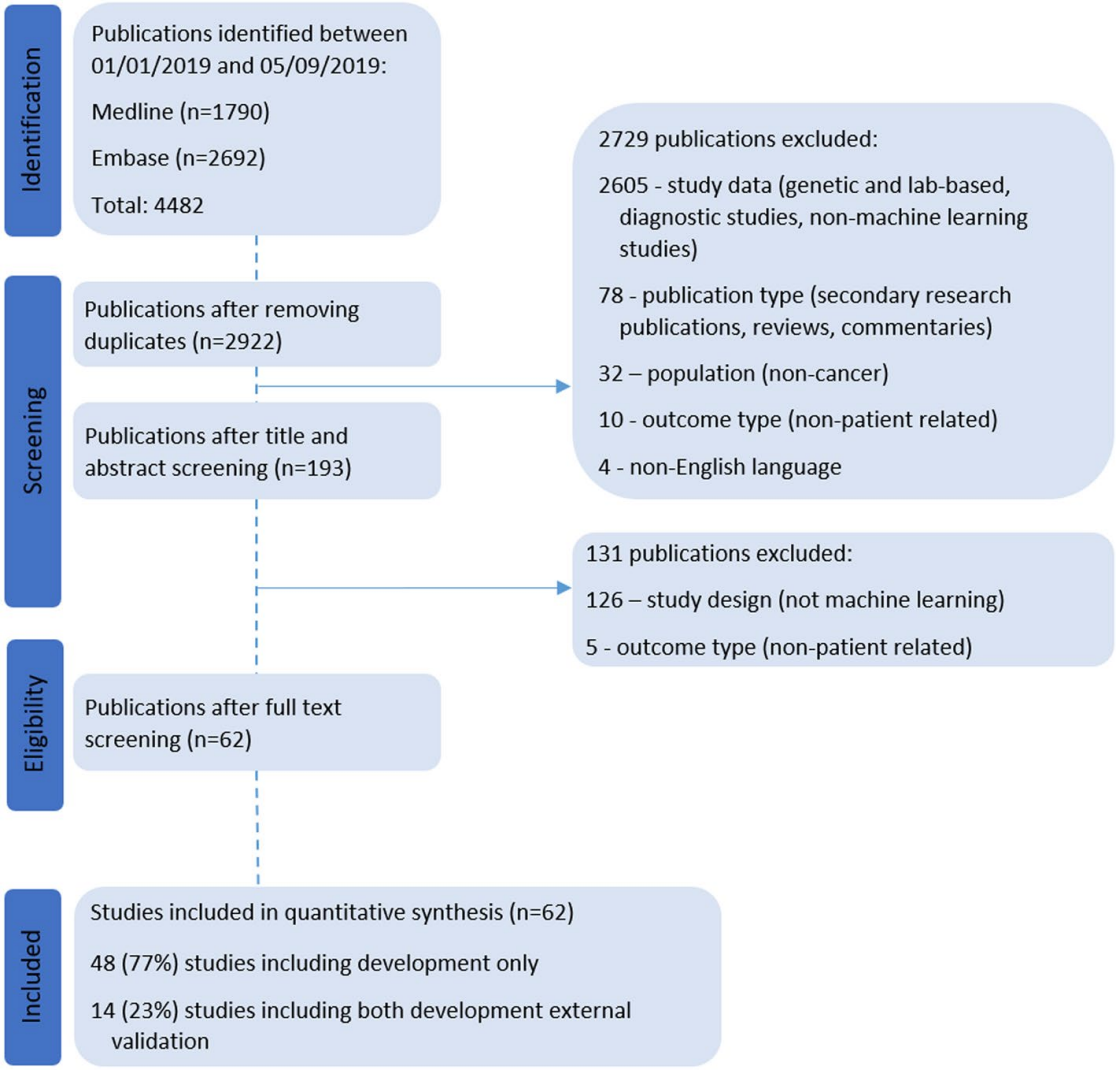
PRISMA flow diagram of included studies

2922 unique studies published between 1 January 2019 and 5 September 2019 were retrieved from MEDLINE and Embase. Title and abstract screening excluded 2729 publications and full text screening excluded a further 131 publication that did not meet the eligibility criteria. 62 publications were included in our review, of which 77% (*n *= 48) were development only studies and 23% (*n* = 14) were development and external validation studies (Fig. 1). Citations for all included studies are provided in Supplementary table 5.

### Study characteristics

The four most prevalent cancers for which models were developed for were lung (*n* = 8, 13%), breast (*n* = 6, 10%), colon (*n* = 6, 10%) and gynaecological cancer (*n* = 6, 8%). The main target population for the developed models were patients diagnosed with cancer (*n* = 55, 89%) and the most common outcomes to be predicted were complications of the cancer such as complications after surgery and clinical leakage (*n* = 11, 18%) and survival as a binary outcome (*n* = 8, 13%), overall survival as a time-to-event outcome (*n* = 7, 11%) and cancer recurrence as a binary outcome (*n* = 7, 11%) (Table 1).

58% of studies developed models in the secondary care setting (36/62), 35% were developed using data from the USA (*n* = 22/62) and 21% were multicentre studies (13/62). More development with validation studies were multicentre studies (*n* = 6/14, 43%) compared to development-only studies (*n* = 7/48, 15%). Developed models were primarily intended for use of healthcare providers (*n* = 40, 65%) but unclear in 31% of studies (*n* = 19) (Table 2).

**Table 2:** Development analysis characteristics of the 62 included publications, by study type

	**All (*n* = 62)**	**Development only (*n* = 48)**	**Development and external validation (*n* = 14)**
	***n* (%)**	***n* (%)**	***n* (%)**
**Development characteristics**			
Data source*			
Randomised controlled trial	1 (1.6)	-	1 (7.1)
Prospective cohort	9 (14.5)	9 (18.8)	-
Retrospective cohort	14 (22.6)	11 (22.9)	3 (21.4)
Registry	21 (33.9)	15 (31.3)	6 (42.9)
Routine care database	9 (14.5)	7 (14.6)	2 (14.3)
Other**	3 (4.8)	2 (4.2)	1 (7.1)
Unclear	5 (8.1)	4 (8.3)	1 (7.1)
Setting***			
Primary care	2 (3.2)	2 (4.2)	-
Secondary care	36 (58.1)	29 (60.4)	7 (50)
Tertiary care	10 (16.1)	7 (14.6)	3 (21.4)
General population	5 (8.1)	3 (6.3)	2 (14.3)
Other****	3 (4.8)	3 (6.3)	-
Unclear	6 (9.7)	4 (8.3)	2 (14.3)
Multicentre*****			
No	26 (41.9)	24 (50)	2 (14.3)
Yes	13 (21)	7 (14.6)	6 (42.9)
Unclear	23 (37.1)	17 (35.4)	6 (42.9)
Geographic location******			
South America	2 (3.2)	2 (4.2)	-
Asia	8 (12.9)	6 (12.5)	2 (14.3)
Europe	13 (21)	13 (27.1)	-
Canada	3 (4.8)	3 (6.3)	-
USA	21 (33.9)	15 (31.3)	6 (42.9)
Europe, North America, Australia	1 (1.6)	1 (2.1)	-
Europe, South America	1 (1.6)	-	1 (7.1)
South Asia, USA	1 (1.6)	1 (2.1)	-
Unclear	12 (19.4)	7 (14.6)	5 (35.7)
**Intended user**			
Health care providers	34 (54.8)	27 (56.3)	7 (50)
Public/patients	2 (3.2)	2 (4.2)	-
Researchers	1 (1.6)	1 (2.1)	-
Health care providers and patient/public	4 (6.5)	1 (2.1)	3 (21.4)
Health care providers and researchers	2 (3.2)	2 (4.2)	-
Unclear	19 (30.6)	15 (31.3)	4 (28.6)
** Aim of model**			
Predict risk	36 (58.1)	25 (52.1)	11 (78.6)
Classify patients	25 (40.3)	23 (47.9)	2 (14.3)
Predict length of stay (continuous outcome)	1 (1.6)	-	1 (7.1)

^*^Validation characteristics for data source are: Randomised controlled trial: 2/14 (14.3%); Prospective cohort: 3/14 (21.4%); Retrospective cohort: 4/14 (28.6%); Registry: 2/14 (14.3%); Routine care database: 2/14 (14.3%); Other (survey): 1/14 (7.1%)

^**^Other includes audit, survey and a combination data source of hospital and research data and a registry

^***^Validation characteristics for setting are: Secondary care: 7/14 (50%); Tertiary care: 4/14 (28.8%); General population: 2/14 (14.3%); Unclear: 1/14 (7.1%)

^****^Other includes combination of hospitals, hospices and nursing homes, NTT medical center in Tokyo and combination of primary and tertiary care

^*****^Validation characteristics for multicentre are: No: 8/14 (57.1%); Yes: 3/14 (21.4%); Unclear: 3/14 (21.4%)

^******^Validation characteristics for geographical location are: South America: 1/14 (7.1%); Asia: 5/14 (35.7%); USA: 5/14 (35.7%); Unclear: 3/14 (21.4%)

Demographic information (*n* = 60, 97%) and tumour characteristics (*n* = 47, 76%) were included as candidate predictors (predictors for potential model inclusion) for most studies. 48% (*n* = 30) used personal history data, 37% (*n* = 23) used physical measurements, 32% (*n* = 20) used blood and urine markers, 27% (*n* = 17) used treatment characteristics, 16% (*n* = 10) used surgery characteristics, 15% (*n* = 9) used patient clinical status and 3% (*n* = 2) used family history as candidate predictors in their models.

A total of 152 prognostic models were developed in the 62 publications. 115 (76%) models were from development-only studies and 37 (24%) were from development with external validation studies. A median of 2 prediction models were developed per publication [range: 1–6] overall and for each study type. Of the 152 developed models, 42 (28%) were regression-based machine learning models (e.g., logistic regression, Cox regression), 71 (47%) were flexible machine learning models (e.g., neural networks, classification and regression trees) and 39 (26%) were ensemble machine learning models (e.g., random forests, gradient boosting machines). Full description of model characteristics is provided in Supplementary table 6.

### Risk of bias

**Fig. 2:**
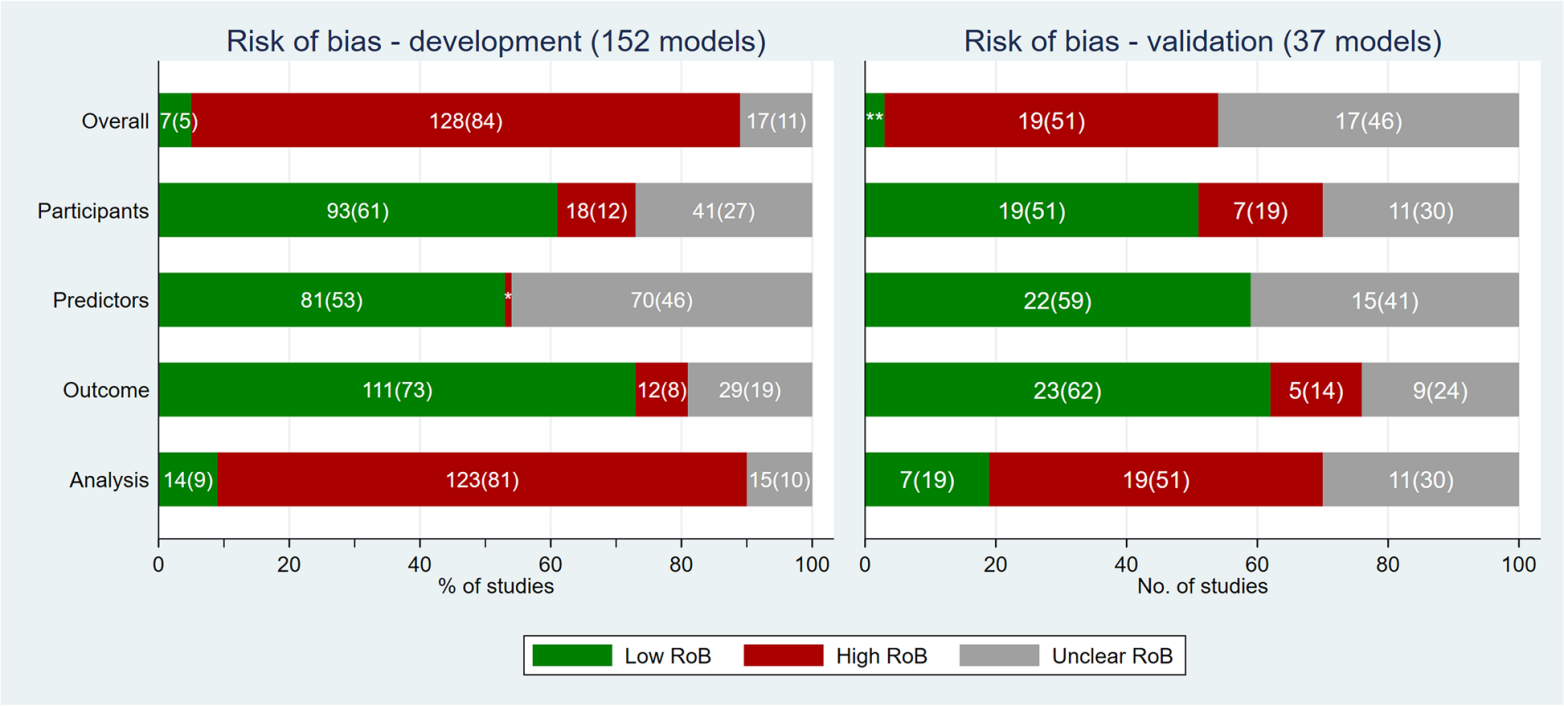
Bar charts showing the risk of bias ratings for each domain and the overall judgement, for the development of 152 models (left) and external validation of 37 developed models (right). “Overall” indicates the overall risk of bias; “participants” indicates bias introduced by participants or data sources; “predictors” indicates bias introduced by predictors or their assessment; “outcome” indicates bias introduced by outcomes or their assessment; “analysis” indicates bias introduced by the analysis. Values in the bars are frequency (%). * values for risk of bias (development models - predictors) are 1(1). ** values for risk of bias (validation models - overall) are 1(3)

Figure 2 summarises the risk of bias judgement for each domain and overall, for each model. 84% (*n* = 128/152; 95% CI: 77 to 89) of the models that were developed were at overall high risk of bias, 5% (*n* = 7/152; 95% CI: 2 to 9) were at low risk of bias and risk of bias was unclear for 11% (*n* = 17/152; 95% CI: 7 to 17) of developed models. 51% (*n* = 19/37; 95% CI: 35 to 67) of models were at overall high risk of bias during external validation and for 46% (*n* = 17/37; 95% CI: 30 to 62) the risk of bias was unclear (due to the information needed to assess risk of bias not being reported or unclear). The validation of only one model was at low risk of bias.

A lower proportion of developed models were at high risk of bias in development with external validation studies (*n* = 28/37; 76%; 95% CI: 59 to 87), compared to development-only studies (*n* = 100/115; 87%; 95% CI: 79 to 92). Overall risk of bias did not differ by model type; development of 83% (*n* = 35/42; 95% CI: 69 to 92) of regression-based models, 85% (*n* = 60/71; 95% CI: 74 to 91) of flexible machine learning models and 85% (*n* = 33/39; 95% CI: 70 to 93) of ensemble models were at high risk of bias. The risk of bias assessment by model type is presented in Supplementary table 7.

In total, information needed for 31% of all signalling questions for risk of bias assessment across the 152 developed models was absent. Information for all model development signalling questions was reported for one model. Sufficient information was reported for a median of 15 (IQR: 13, 16.5, range: 2 to 20) model development signalling questions. Similarly, information needed for 33% of signalling questions for risk of bias assessment across the 37 validation analyses was absent. Information for all model validation signalling questions was reported for no models. Sufficient information was reported for a median of 13 (IQR: 10, 14, range: 2 to 16) model validation signalling questions.

A median of 4 signalling questions (IQR: 3, 5, range: 0–10) were linked to a high risk of bias (answered ‘No/Probably No’ (‘N/PN’)) in development analyses and a median of 1 domain was at high risk of bias (IQR: 1, 1, range 0–4). A median of 2 signalling questions (IQR: 1, 2, range: 1–3) were linked to a high risk of bias (answered ‘No/Probably No’ (‘N/PN’)) in validation analyses and a median of 1 domain was at high risk of bias (IQR: 0, 1, range 0–3).

### Participants domain

Risk of bias introduced by the participants domain was low for the development and validation analyses of 61% (*n* = 93/152; 95% CI: 53 to 69) and 51% (*n* = 19/37; 95% CI: 35 to 67) of models, respectively. Data sources (signalling question 1.1) were considered appropriate for 76% (*n* = 115/152; 95% CI: 68 to 82) developed models and 81% (*n* = 30/37; 95% CI: 65 to 91) validated models (Table 3). Inclusion and exclusion of participants (signalling question 1.2) was not reported or unclear in over a quarter of developed models (*n *= 39/152; 25%, 95% CI: 19 to 33) and over a third of validated models (*n* = 13/37; 35%, 95% CI: 21 to 52).

**Table 3:** PROBAST signalling questions for model development and validation analyses in all 62 studies

PROBAST domain and signalling questions	Development analysis (152 models)			Validation analysis (37 models)		
	**Yes/probably yes**	**No/probably no**	**No information**	**Yes/probably yes**	**No/probably no**	**No information**
	**n (%; 95% CI)**	**n (%; 95% CI)**	**n (%; 95% CI)**	**n (%; 95% CI)**	**n (%; 95% CI)**	**n (%; 95% CI)**
1. PARTICIPANTS						
1.1. Were appropriate data sources used, e.g., cohort, randomized controlled trial, or nested case–control study data?	115 (75.7; 68.1,81.9)	19 (12.5; 8.1,18.8)	18 (11.8; 7.6,18.1)	30 (81.1; 64.7,90.9)	2 (5.4; 1.3,20)	5 (13.5; 5.6.29.3)
1.2. Were all inclusions and exclusions of participants appropriate?	100 (65.8; 57.8,72.9)	13 (8.6; 5,14.2)	39 (25.7; 19.3,33.3)	24 (64.9; 47.9,78.8)	-	13 (35.1; 21.2.52.1)
2. PREDICTORS						
2.1. Were predictors defined and assessed in a similar way for all participants?	117 (77; 69.6,83)	14 (9.2; 5.5,15)	21 (13.8; 9.2,20.3)	26 (70.3; 53.3,83.1)	-	11 (29.7; 16.9.46.7)
2.2. Were predictor assessments made without knowledge of outcome data?	73 (48; 40.1,56)	1 (0.7; 0.1,4.6)	78 (51.3; 43.3,59.2)	20 (54.1; 37.6,69.7)	-	17 (46; 30.3.62.4)
2.3. Are all predictors available at the time the model is intended to be used?	91 (59.9; 51.8,67.4)	-	61 (40.1; 32.6,48.2)	22 (59.5; 42.7,74.3)	-	15 (40.5; 25.7.57.3)
3. OUTCOMES						
3.1. Was the outcome determined appropriately?	130 (85.5; 78.9,90.3)	4 (2.6; 1,6.9)	18 (11.8; 7.6,18.1)	30 (81.1; 64.7,90.9)	-	7 (18.9; 9.1.35.3)
3.2. Was a prespecified or standard outcome definition used?	122 (80.3; 73.1,85.9)	13 (8.6; 5,14.2)	17 (11.2; 7,17.3)	23 (62.2; 45.2,76.6)	7 (18.9; 9.1,35.3)	7 (18.9; 9.1.35.3)
3.3. Were predictors excluded from the outcome definition?	117 (77; 69.6,83)	6 (4; 1.8,8.6)	29 (19.1; 13.6,26.2)	28 (75.7; 58.9,87.1)	-	9 (24.3; 12.9.41.1)
3.4. Was the outcome defined and determined in a similar way for all participants?	115 (75.7; 68.1,81.9)	11 (7.2; 4,12.6)	26 (17.1; 11.9,24)	35 (94.6; 80,98.7)	-	2 (5.4; 1.3.20)
3.5. Was the outcome determined without knowledge of predictor information?	106 (69.7; 61.9,76.6)	6 (4; 1.8,8.6)	40 (26.3; 19.9,33.9)	28 (75.7; 58.9,87.1)	-	9 (24.3; 12.9.41.1)
3.6. Was the time interval between predictor assessment and outcome determination appropriate?	100 (65.8; 57.8,72.9)	5 (3.3; 1.4,7.7)	47 (30.9; 24,38.8)	21 (56.8; 40.1,72)	5 (13.5; 5.6,29.3)	11 (29.7; 16.9.46.7)
4. ANALYSIS						
4.1. Were there a reasonable number of participants with the outcome?	44 (29; 22.2,36.7)	77 (50.7; 42.7,58.6)	31 (20.4; 14.7,27.6)	10 (27; 14.9,44)	16 (43.2; 28,59.9)	11 (29.7; 16.9,46.7)
4.2. Were continuous and categorical predictors handled appropriately?	30 (19.7; 14.1,26.9)	57 (37.5; 30.1,45.5)	65 (42.8; 35.1,50.8)	19 (51.4; 35.1,67.3)	1 (2.7; 0.4,17.8)	17 (46; 30.3,62.4)
4.3. Were all enrolled participants included in the analysis?	43 (28.3; 21.7,36)	49 (32.2; 25.2,40.1)	60 (39.5; 32,47.5)	17 (46; 30.3,62.4)	9 (24.3; 12.9,41.1)	11 (29.7; 16.9,46.7)
4.4. Were participants with missing data handled appropriately?	24 (15.8; 10.8,22.5)	70 (46.1; 38.2,54.1)	58 (38.2; 30.7,46.2)	6 (16.2; 7.3,32.4)	15 (40.5; 25.7,57.3)	16 (43.2; 28,59.9)
4.5. Was selection of predictors based on univariable analysis avoided?	68 (44.7; 37,52.8)	49 (32.2; 25.2,40.1)	35 (23; 17,30.4)	NA		
4.6. Were complexities in the data (e.g., censoring, competing risks, sampling of control participants) accounted for appropriately?	10 (6.6; 3.6,11.8)	28 (18.4; 13,25.5)	114 (75; 67.4,81.3)	2 (5.4; 1.3,20)	-	35 (94.6; 80,98.7)
4.7. Were relevant model performance measures evaluated appropriately?	28 (18.4; 13,25.5)	87 (57.2; 49.2,64.9)	37 (24.3; 18.1,31.9)	10 (27; 14.9,44)	13 (35.1; 21.2,52.1)	14 (37.8; 23.4,54.8)
4.8. Were model overfitting and optimism in model performance accounted for?	52 (34.2; 27.1,42.2)	84 (55.3; 47.2,63)	16 (10.5; 6.5,16.5)	NA		
4.9. Do predictors and their assigned weights in the final model correspond to the results from the reported multivariable analysis?	24 (15.8; 10.8,22.5)	8 (5.3; 2.6,10.2)	120 (79; 71.7,84.7)	NA		

*Y*  Yes, *PY*  Probably yes, *N*  No, *PN*  Probably no, *NI*  No information

### Predictors domain

Risk of bias introduced by the predictors domain was unclear for the development and validation of 46% (*n* = 70/152; 95% CI: 38 to 54) and 41% (*n* = 15/37; 95% CI: 26 to 57) of models, respectively. The largest contributors to this unclear risk of bias were blinding of predictor assessments to the outcome (signalling question 2.2) and availability of predictors at the time of intended use (signalling question 2.3). Insufficient information about blinding of predictors was reported for development of 51% (78/152; 95% CI: 43 to 59) and validation of 46% (*n* = 17/37; 95% CI: 30 to 62) of models. The availability of predictors and timing of intended use was unclear or not reported for 40% of both model development (*n* = 61/152; 95% CI: 33 to 48) and validation (*n* = 15/37; 95% CI: 267 to 57).

### Outcome domain

Risk of bias introduced by the outcome domain was low for the development and validation of 73% (*n* = 111/152; 95% CI: 65 to 80) and 62% (*n* = 23/37; 95% CI: 45 to 77) of models, respectively. Objective outcome measures such as survival and mortality were used in 35% (*n* = 22/62) of studies. Over 75% of models were developed using an appropriately determined outcome (signalling question 3.1), pre-specified or standard outcome definition (signalling question 3.2), excluding predictor information (signalling question 3.3) and were defined in a similar way for all participants (signalling question 3.4).

Similar to the predictor domain, unclear risk of bias was introduced because of no information reported about the blinding of the outcome (signalling question 3.5) and the timing between predictor assessment and outcome determination (signalling question 3.6) for both development and validation analyses. For example, information about timing between predictor assessment and outcome determination was lacking in 31% (*n* = 47/152; 95% CI: 24 to 39) and 30% (*n* = 11/37; 95% CI: 17 to 47) of models.

### Analysis domain

Comprising 9 signalling questions, bias introduced in the analysis was the largest contributor to the overall risk of bias judgement for model development and validation. 81% (*n* = 123/152; 95% CI: 74 to 86) models were judged at high risk of bias in the analysis domain when developed, and 51% (*n* = 19/37; 95% CI: 35 to 67) models were at high risk on bias when validated.

Half of models had concerns of using an insufficient sample size because there were less than 10 events-per-predictor (and no other justification for why overfitting would be minimised with the sample size) (*n* = 77/152; 51%, 95% CI: 43 to 57) and no information (i.e., missing number of events or number of candidate predictors) was reported for a further 20% of models (*n* = 31/152; 95% CI: 15 to 28). Of the 28 studies (*n* = 28/62, 45%) reporting both the number of outcome events and number of candidate predictors used for model development, a median of 9.4 events per predictor was used (IQR: 1.7, 21.3, range: 0.2 to 5836.5).

Handling missing data was considered inadequate for 46% (*n* = 70/152; 95% CI: 38 to 54) of developed models and for a further 38% (*n* = 58/152; 95% CI: 31 to 46) of models, no information on either the presence or handling of missing data was reported. Twenty-four studies (39%) reported methods to handle missing data and complete case analysis was the most common method (*n* = 10/24, 41%). Mean, median, or mode imputation was used in six studies (*n* = 6/24, 25%) and multiple imputation was used in five studies (*n* = 5/24, 21%). Two studies used subsequent follow-up data to complete the missing data and another study used a k-nearest neighbour algorithm.

Selection of predictors was informed by their univariable association with the outcome for 32% of models (*n* = 49/152), and thus signalling high risk of bias due to concerns of inappropriate selection. No information about methods to handle complexities of the data (e.g., censoring and competing risks) was reported for 75% (*n* = 114/152; 95% CI: 67 to 81) of developed and 95% (*n* = 35/37; 95% CI: 80 to 99) of validated models.

Few models were appropriately evaluated using relevant model performance measures (e.g., discrimination and calibration) during model development and validation (*n* = 28/152; 18%, 95% CI: 13 to 26 and *n* = 10/37; 27%, 95% CI: 15 to 44, respectively). Discrimination performance measures (e.g., area under the curve) and other performance measures (sensitivity, specificity) were often reported during development (*n* = 47/62; 76% and *n* = 43/62; 69% of studies, respectively) and validation analyses (*n* = 11/14; 77% and *n* = 7/14; 50% of studies, respectively); but assessment of calibration of observed and predicted risks (e.g., via a calibration plot) was often missing in both analyses (*n* = 51/62; 82%; and *n* = 8/14; 57%, respectively).

Few models accounted for possible over- and under- fitting and optimism (34%, 95% CI: 27 to 42) with a split sample approach as the most popular method to internally validate developed models (45% of studies). No information was found for 79% of models regarding their assigned weights in the final model and their correspondence to the results of the main analysis.

A full description of the risk of bias assessment for each included study is provided on the Open Science Framework (osf.io/95ayc/) [35].

## Discussion

### Summary of findings

In this systematic review we assessed the risk of bias of prognostic model development and validation studies that applied author-defined machine learning methods. We used a formal risk of bias tool and found studies were judged at high risk of bias for nearly all the models included in the review. Fewer validation analyses were at high risk of bias but more had an unclear risk of bias, due to poor and incomplete reporting. No difference was observed in the risk of bias between the different types of machine learning modelling approaches.

The analysis domain was the largest contributor to the high risk of bias amongst models, where insufficient sample size, categorisation of predictors, exclusion of missing data, split sample internal validation, poor reporting of relevant model performance measures (discrimination and calibration), and lack of information for the full (final) model were the main reasons. Few models also appropriately handled complexities in the data, for example, censoring was not accounted for in most models developed for a time-to-event outcome. Model performance measures were often discrimination and classification performance measures and calibration measures were rarely reported.

### Literature

There is a paucity of research systematically evaluating the risk of bias and details of factors contributing to increased risk of bias for machine learning based clinical prediction models. Existing reviews in oncology have largely focused on models from specific medical sub-specialties and cancer types in which very few machine learning models are included and general modelling inferences are made [15, 36–39]. However, our findings are in line with the general findings of these reviews that models developed are often poor in methodological quality and at high risk of bias.

Our systematic review also supports evidence which has been highlighted by more general reviews of machine learning based prediction models [18, 21, 40–43]. Christodoulou et al. compared logistic regression to machine learning prediction models and observed many studies at a high risk of bias, those at high risk of bias tended to erroneously favour machine learning, and highlighted a need to follow reporting and methodological guidance [18]. Sufriyana et al. conducted a review about models in pregnancy care and also judged most models at high risk of bias, with negligible differences in risk of bias between regression-based and machine learning modelling approaches [42]. Shung et al. reviewed models to predict outcomes in patients with gastrointestinal bleeding, and though they found good performance in these models, all were at high risk of bias, evaluated using the Quality In Prognosis Studies [44] casting doubt on their credibility and usefulness.

Insufficient sample size when developing and validating machine learning based prediction models is a major design flaw and contributor to risk of bias [18, 19, 22]. We observed instances where sample size for (non-regression based) machine learning models was based on sample size considerations for regression-based prediction model studies. However, studies have shown that much larger sample sizes are needed when using machine learning methods and so the impact and risk of bias introduced from these insufficient sample sizes may be much larger [45, 46], as PROBAST is primarily aimed at regression-based prediction models [30, 31].

### Strengths and limitations

This review highlights the methodological flaws and factors contributing to the high risk of bias associated with machine learning based clinical prediction models in oncology. Where existing systematic reviews have focussed on the quality of models in certain clinical sub-specialties and cancer types, we provide a broader view that focusses on the formal risk of bias assessment using PROBAST of prediction model studies using author defined machine learning methods across oncology.

We searched MEDLINE and Embase, two major biomedical literature databases for studies that developed (and validated) a machine learning based clinical prediction model. However, we may have missed eligible publications. We also restricted our search to models that were published during 01 Jan 2019 and 05 Sept 2019 and given the high rate of publication in this field, we will have missed models published since our search date. However, for this study we aimed to review a contemporary sample of publications to reflect current practice. Further, given the agreement between our findings and existing evidence, it is unlikely that additional studies would change the conclusion of this review.

We used the formal PROBAST risk of bias tool for assessing diagnostic and prognostic prediction models [30, 31]. We evaluated 152 models from 62 studies, with many studies developing and validating more than one model, in these instances we assumed the same risk of bias profile across all models that were developed, unless further model specific information was provided. This may limit our study by discounting machine learning models for which, methods of handling missing data, continuous predictors and other complexities of the data, are implicit to their analytical approach. However, we believe this is a fair assumption, especially for the ‘participants’, ‘predictors’ and ‘outcomes’ domain which evaluates study design factors such as participant eligibility criteria and outcome and predictor definitions which are unlikely change with the modelling methods.

The risk of bias profile may differ in the analysis domain and in particular for signalling question 4.2. ‘Were continuous and categorical predictors handled appropriately?’ Here, we remained conservative in our assumption as we found some studies that reported the analytical methods, despite using flexible machine learning models and did not want to assume the reverse (if a flexible machine learning model was developed, continuous predictors were by default modelled appropriately).

Although PROBAST was primarily designed for regression-based prediction, the majority of the 20 signalling items are applicable to machine learning based prediction model studies. No other risk of bias tool is yet available specifically for machine learning models. Some items in PROBAST may indeed be less straightforward to assess for machine learning methods (e.g., ‘do predictors and their assigned weights in the final model correspond to the results from the reported multivariable analysis?’), but all other items are applicable. It is also more likely that some relevant risk of bias items specific for machine learning models may be missing and that the overall risk of bias of these models are even poorer than what was found.

### Future research

The Transparent Reporting of a multivariable prediction model for Individual Prognosis Or Diagnosis (TRIPOD) collaboration has initiated the development of a TRIPOD statement specific to machine learning (TRIPOD-AI) to improve reporting conduct [47–49]. A similar initiative has also started to develop a specific PROBAST-AI tool for diagnostic and prognostic prediction models developed using machine learning that includes additional items to evaluate to analysis and presentation on machine learning models. Based on items leading to high risk of bias in machine learning models, urgent methodological guidance is needed to support researchers developing clinical prediction models using machine learning to ensure use better and efficient modelling methods. There is a particular need for sample size guidance that will ensure informed and justified use to data to develop these models.

Given the rapid and evolving nature of the machine learning based clinical prediction models in oncology, periodic reviews and re-reviews are needed so evidence reflects current practice. These reviews should both focus on individual clinical domains and be cancer specific but should also focus on machine learning based prediction models.

## Conclusions

The quality of machine learning based prognostic models is poor in oncology, and hence most models have a high risk of bias, limiting their usefulness in daily practice. Particular attention and methodological guidance is needed for sample size estimation and analysis methods to improve quality of machine learning based clinical prediction models. Researchers should improve the availability and reporting of developed models so they can be independently evaluated and considered to clinical practice. The reported performance of existing models should be interpreted with caution given their high risk of bias.

## Supplementary Information

**Additional file 1. **Supplementary information 

## Data Availability

Data that has informed the analysis, can be found on the Open Science Framework (https://osf.io/95ayc/).

## Abbreviations

TRIPOD: Transparent Reporting of a multivariable prediction model for Individual Prognosis Or Diagnosis
PROBAST: Prediction model Risk Of Bias ASsessment Tool
