# Supplementary material for: Risk of bias of prognostic models developed using machine learning: a systematic review in oncology
Source: Diagn Progn Res. 2022 Jul 7;6:13. doi: 10.1186/s41512-022-00126-w (PMC9261114; doi:10.1186/s41512-022-00126-w)
Supplement: Supplementary file 1 — Additional file 1. Supplementary information [file 41512_2022_126_MOESM1_ESM.docx]

**Supplementary information**

**Supplementary table 1 - MEDLINE search strategy**

Database and platform: MEDLINE (Ovid MEDLINE® Epub Ahead of Print, In-Process & Other Non-Indexed Citations, Ovid MEDLINE® Daily and Ovid MEDLINE®) 1946 to present (via Ovid)

Publication date limit: 2019 only

Search date: 5 September 2019

| 1 | Machine Learning/ |
| --- | --- |
| 2 | (machine adj1 (learn$ or model$)).ti,ab,kw. |
| 3 | Deep Learning/ |
| 4 | (deep adj2 learn$).ti,ab,kw. |
| 5 | exp Supervised Machine Learning/ |
| 6 | (supervised adj2 machine adj2 learn$).ti,ab,kw. |
| 7 | ((support or relevance) adj2 vector adj2 (machine$ or classification$)).ti,ab,kw. |
| 8 | "Neural Networks (Computer)"/ |
| 9 | (neural adj2 network$).ti,ab,kw. |
| 10 | ((statistical or "statistical-learning") adj1 (learn$ or strateg$)).ti,ab.kw. |
| 11 | (multi adj2 layer adj1 perceptron$).ti,ab,kw. |
| 12 | (random adj2 forest$).ti,ab,kw. |
| 13 | "RF classifi$".ti,ab,kw. |
| 14 | (lasso or ridge or kernel or ensemble or bagging or bagged or bootstrap$ or boosting or boosted or fuzzy).ti,ab,kw. |
| 15 | ((penali?ed or regulari?ed) adj2 ('likelihood' or 'regression' or 'logistic' or 'survival' or 'estimat$' or 'function$' or 'method$' or 'least' or 'ensemble')).ti,ab,kw. |
| 16 | ((classification or regression or estimation or decision) adj2 tree$).ti,ab,kw. |
| 17 | (bayes$ adj1 network$).ti,ab,kw. |
| 18 | (nearest adj1 neighbo?r).ti,ab,kw. |
| 19 | (k-nearest adj1 neighbo?r).ti,ab,kw. |
| 20 | (elastic adj1 net).ti,ab,kw. |
| 21 | (naive adj1 bayes$).ti,ab,kw. |
| 22 | ((nonparametric or "non-parametric") adj2 (model$ or analys$)).ti,ab,kw |
| 23 | (KNN or ANN or ANNs or RNN or RF or SVM or NB or CART or DT or MLP).ti,ab,kw. |
| 24 | or/1-23 |
| 25 | Logistic Models/ |
| 26 | (logistic adj2 (model$ or regression)).ti,ab,kw. |
| 27 | Linear Models/ |
| 28 | (linear adj2 (model$ or regression)).ti,ab,kw. |
| 29 | (proportion$ adj2 odds adj2 regression).ti,ab,kw. |
| 30 | Least-Squares Analysis/ |
| 31 | (least adj2 square$).ti,ab,kw. |
| 32 | Survival Analysis/ |
| 33 | (survival adj1 (analys$ or model$)).ti,ab,kw. |
| 34 | Proportional Hazards Models/ |
| 35 | (proportional adj1 hazard$).ti,ab,kw. |
| 36 | ((cox or parametric) adj1 (regression or model$)).ti,ab,kw. |
| 37 | (semi adj2 parametric adj1 (regression or model$)).ti,ab,kw. |
| 38 | Disease-Free Survival/ |
| 39 | Progression-Free Survival/ |
| 40 | ((disease or progression or event) adj2 free adj1 survival).ti,ab,kw. |
| 41 | (overall adj1 survival).ti,ab,kw. |
| 42 | or/25-41 |
| 43 | 24 and 42 |
| 44 | 24 or 43 |
| 45 | exp Neoplasms/ |
| 46 | (cancer$ or tumour$ or tumor$ or carcinoma$ or malignan$ or neoplas$ or sarcoma$ or adenocarcinoma$ or carcinogen$ or metasta$ or oncolog$).ti,ab,kw. |
| 47 | or/45-46 |
| 48 | Prognosis/ |
| 49 | (prognos$ adj1 (modelling or modeling or model or models or predict$ or index or performance or nomogram or tools or ability or accuracy or probability or risk or factor$ or marker$ or biomarker$)).ti,ab,kw. |
| 50 | "risk model$".ti,ab,kw. |
| 51 | "predict$ the prognosis of".ti,ab,kw. |
| 52 | "predict$ the risk of".ti,ab,kw. |
| 53 | "predict$ the probability of".ti,ab,kw. |
| 54 | Probability/ |
| 55 | (probability adj1 (modelling or modeling or model or models)).ti,ab,kw. |
| 56 | (predict$ adj1 (modelling or modeling or model or models or nomogram or tools or performance or ability or index or accuracy or probability or risk or factor$ or marker$ or biomarker$)).ti,ab,kw. |
| 57 | "candidate predictor$".ti,ab,kw. |
| 58 | "predictive clinical parameter$".ti,ab,kw. |
| 59 | ((discrimination or discriminative or discriminatory) adj1 (accuracy or ability or performance or value or model or models or power or capacity or capabilit$ or efficiency)).ti,ab,kw. |
| 60 | (discriminability or c-index or c-statistic or concordance or DCA).ti,ab,kw. |
| 61 | "decision curve".ti,ab,kw. |
| 62 | (calibrat$ adj1 (plot$ or curve$ or slope$ or model or models)).ti,ab,kw. |
| 63 | (brier adj1 score$).ti,ab,kw. |
| 64 | (performance adj1 (classification or classifier or clinical or accuracy or validation or metrics or diagnostic or AUC)).ti,ab,kw. |
| 65 | (sensitivity or specificity or PPV or NPV).ti,ab,kw. |
| 66 | "correctly classified".ti,ab,kw. |
| 67 | "clinical accuracy".ti,ab,kw. |
| 68 | "positive predictive value$".ti,ab,kw. |
| 69 | "negative predictive value$".ti,ab,kw. |
| 70 | (classification or classifier).ti,ab,kw. |
| 71 | Area Under Curve/ |
| 72 | "Area under the curve".ti,ab,kw. |
| 73 | "Area under the ROC curve".ti,ab,kw. |
| 74 | "Area under the ROC".ti,ab,kw. |
| 75 | "Area Under the Receiver Operat$ Characteristic$".ti,ab,kw. |
| 76 | ROC Curve/ |
| 77 | "receiver operating characteristic$".ti,ab,kw. |
| 78 | (ROC or AUC or AUROC).ti,ab,kw. |
| 79 | "Hosmer-Lemeshow".ti,ab,kw. |
| 80 | "H-L test".ti,ab,kw. |
| 81 | "expected ratio".ti,ab,kw. |
| 82 | "observed ratio".ti,ab,kw. |
| 83 | "E:O ratio".ti,ab,kw. |
| 84 | or/48-83 |
| 85 | 44 and 47 and 84 |
| 86 | Limit 85 to yr="2019" |

**Supplementary table 2 - EMBASE search strategy**

Database and platform: Embase 1974 to present (via Ovid)

Publication d limit: 2019 only

Search date: 5 September 2019

| 1 | exp Machine Learning/ |
| --- | --- |
| 2 | (machine adj1 (learn$ or model$)).ti,ab,kw. |
| 3 | (deep adj2 learn$).ti,ab,kw. |
| 4 | (supervised adj2 machine adj2 learn$).ti,ab,kw. |
| 5 | ((support or relevance) adj2 vector adj2 (machine$ or classification$)).ti,ab,kw. |
| 6 | (neural adj2 network$).ti,ab,kw. |
| 7 | ((statistical or "statistical-learning") adj1 (learn$ or strateg$)).ti,ab,kw. |
| 8 | (multi adj2 layer adj1 perceptron$).ti,ab,kw. |
| 9 | (random adj2 forest$).ti,ab,kw. |
| 10 | "RF classifi$".ti,ab,kw. |
| 11 | Bootstrapping/ |
| 12 | (lasso or ridge or kernel or ensemble or bagging or bagged or bootstrap$ or boosting or boosted or fuzzy).ti,ab,kw. |
| 13 | ((penali?ed or regulari?ed) adj2 (likelihood or regression or logistic or survival or estimat$ or function$ or method$ or least or ensemble)).ti,ab,kw. |
| 14 | Decision Tree/ |
| 15 | ((classification or regression or estimation or decision) adj2 tree$).ti,ab,kw. |
| 16 | (naive adj1 bayes$).ti,ab,kw. |
| 17 | (bayes$ adj1 network$).ti,ab,kw. |
| 18 | (nearest adj1 neighbo?r).ti,ab,kw. |
| 19 | (k-nearest adj1 neighbo?r).ti,ab,kw. |
| 20 | (elastic adj1 net).ti,ab,kw. |
| 21 | Nonparametric Test/ |
| 22 | ((nonparametric or "non-parametric") adj2 (model$ or analys$)).ti,ab,kw. |
| 23 | (KNN or ANN or ANNs or RNN or RF or SVM or NB or CART or DT or MLP).ti,ab,kw. |
| 24 | or/1-23 |
| 25 | Logistic Regression Analysis/ |
| 26 | (logistic adj2 (model$ or regression)).ti,ab,kw. |
| 27 | Linear Regression Analysis/ |
| 28 | (linear adj2 (model$ or regression)).ti,ab,kw. |
| 29 | (proportion$ adj2 odds adj2 regression).ti,ab,kw. |
| 30 | Least Square Analysis/ |
| 31 | (least adj2 square$).ti,ab,kw. |
| 32 | Survival Analysis/ |
| 33 | (survival adj1 (analys$ or model$)).ti,ab,kw. |
| 34 | Proportional Hazards Models/ |
| 35 | (proportional adj1 hazard$).ti,ab,kw. |
| 36 | ((cox or parametric) adj1 (regression or model$)).ti,ab,kw. |
| 37 | (semi adj2 parametric adj1 (regression or model$)).ti,ab,kw. |
| 38 | Disease-Free Survival/ |
| 39 | Progression-Free Survival/ |
| 40 | ((disease or progression or event) adj2 free adj1 survival).ti,ab,kw. |
| 41 | (overall adj1 survival).ti,ab,kw. |
| 42 | or/25-41 |
| 43 | 24 and 42 |
| 44 | 24 or 43 |
| 45 | exp Neoplasm/ |
| 46 | (cancer$ or tumour$ or tumor$ or carcinoma$ or malignan$ or neoplas$ or sarcoma$ or adenocarcinoma$ or carcinogen$ or metasta$ or oncolog$).ti,ab,kw. |
| 47 | or/45-46 |
| 48 | exp Prognosis/ |
| 49 | (prognos$ adj1 (modelling or modeling or model or models or predict$ or index or performance or nomogram or tools or ability or accuracy or probability or risk or factor$ or marker$ or biomarker$ or parameter$)).ti,ab,kw. |
| 50 | "risk model$".ti,ab,kw. |
| 51 | "predict$ the prognosis of".ti,ab,kw. |
| 52 | "predict$ the risk of".ti,ab,kw. |
| 53 | "predict$ the probability of".ti,ab,kw. |
| 54 | (probability adj1 (modelling or modeling or model or models)).ti,ab,kw. |
| 55 | Prediction/ |
| 56 | (predict$ adj1 (modelling or modeling or model or models or nomogram or tools or performance or ability or index or accuracy or probability or risk or factor$ or marker$ or biomarker$)).ti,ab,kw. |
| 57 | "candidate predictor$".ti,ab,kw. |
| 58 | "predictive clinical parameter$".ti,ab,kw. |
| 59 | ((discrimination or discriminative or discriminatory) adj1 (accuracy or ability or performance or value or model or models or power or capacity or capabilit$ or efficiency)).ti,ab,kw. |
| 60 | (discriminability or c-index or c-statistic or concordance or DCA).ti,ab,kw. |
| 61 | "decision curve".ti,ab,kw. |
| 62 | Calibration/ |
| 63 | (calibrat$ adj1 (plot$ or curve$ or slope$ or model or models)).ti,ab,kw. |
| 64 | (brier adj1 score$).ti,ab,kw. |
| 65 | (performance adj1 (classification or classifier or clinical or accuracy or validation or metrics or diagnostic or AUC)).ti,ab,kw. |
| 66 | Validation Process/ |
| 67 | (sensitivity or specificity or PPV or NPV).ti,ab,kw. |
| 68 | "correctly classified".ti,ab,kw. |
| 69 | (classification or classifier).ti,ab,kw. |
| 70 | "clinical accuracy".ti,ab,kw. |
| 71 | "positive predictive value$".ti,ab,kw. |
| 72 | "negative predictive value$".ti,ab,kw. |
| 73 | Predictive value/ |
| 74 | Probability/ |
| 75 | "Area Under the Curve"/ |
| 76 | "Area Under the Curve Ratio"/ |
| 77 | "Area under the curve".ti,ab,kw. |
| 78 | "Area under the ROC curve".ti,ab,kw. |
| 79 | "Area under the ROC".ti,ab,kw. |
| 80 | "Area Under the Receiver Operat$ Characteristic$".ti,ab,kw. |
| 81 | ROC Curve/ |
| 82 | Receiver Operating Characteristic/ |
| 83 | "receiver operating characteristic$".ti,ab,kw. |
| 84 | (ROC or AUC or AUROC).ti,ab,kw. |
| 85 | "Hosmer-Lemeshow".ti,ab,kw. |
| 86 | "H-L test".ti,ab,kw. |
| 87 | "expected ratio".ti,ab,kw. |
| 88 | "observed ratio".ti,ab,kw. |
| 89 | "E:O ratio".ti,ab,kw. |
| 90 | or/48-89 |
| 91 | 44 and 47 and 90 |
| 92 | conference abstract.pt. |
| 93 | conference abstract.st. |
| 94 | 92 or 93 |
| 95 | 91 not 94 |
| 96 | Limit 95 to yr="2019" |

**Supplementary table 3.** PROBAST domains and signalling questions used for data extraction [30,31].

| **PROBAST domain and signalling questions** | **Development analysis** | **Validation analysis** |
| --- | --- | --- |
| 1. **PARTICIPANTS** |  |  |
| - 1. *Were appropriate data sources used, e.g., cohort, randomized controlled trial, or nested case–control study data?* | ✓ | ✓ |
| - 1. *Were all inclusions and exclusions of participants appropriate?* | ✓ | ✓ |
| PREDICTORS |  |  |
| - 1. *Were predictors defined and assessed in a similar way for all participants?* | ✓ | ✓ |
| - 1. *Were predictor assessments made without knowledge of outcome data?* | ✓ | ✓ |
| - 1. *Are all predictors available at the time the model is intended to be used?* | ✓ | ✓ |
| 1. **OUTCOMES** |  |  |
| - 1. *Was the outcome determined appropriately?* | ✓ | ✓ |
| - 1. *Was a prespecified or standard outcome definition used?* | ✓ | ✓ |
| - 1. *Were predictors excluded from the outcome definition?* | ✓ | ✓ |
| - 1. *Was the outcome defined and determined in a similar way for all participants?* | ✓ | ✓ |
| - 1. *Was the outcome determined without knowledge of predictor information?* | ✓ | ✓ |
| - 1. *Was the time interval between predictor assessment and outcome determination appropriate?* | ✓ | ✓ |
| 1. **ANALYSIS** |  |  |
| - 1. *Were there a reasonable number of participants with the outcome?* | ✓ | ✓ |
| - 1. *Were continuous and categorical predictors handled appropriately?* | ✓ | ✓ |
| - 1. *Were all enrolled participants included in the analysis?* | ✓ | ✓ |
| - 1. *Were participants with missing data handled appropriately?* | ✓ | ✓ |
| - 1. *Was selection of predictors based on univariable analysis avoided?* | ✓ | 🗶 |
| - 1. *Were complexities in the data (e.g., censoring, competing risks, sampling of control participants) accounted for appropriately?* | ✓ | ✓ |
| - 1. *Were relevant model performance measures evaluated appropriately?* | ✓ | ✓ |
| - 1. *Were model overfitting and optimism in model performance accounted for?* | ✓ | 🗶 |
| - 1. *Do predictors and their assigned weights in the final model correspond to the results from the reported multivariable analysis?* | ✓ | 🗶 |

**Supplementary table 4. PROBAST risk of bias assessment**

| **Participants** | |
| --- | --- |
| **1.1 Were appropriate data sources used, e.g. cohort, RCT or nested case-control study?** | **Decreases ROB (Y/PY)**   - prospective longitudinal cohorts (RCT or proper registry) with consistent methods for participant inclusion and exclusion – predefined predictors and outcome determination - case control/cohort studies are low ROB if the original cohort/registry outcome frequency is adjusted for – look for ‘reweighting’ or ‘inverse sampling fraction’ of the outcome.   **Increases ROB (N/PN)**   - existing cohorts with potentially inconsistent participant inclusion/exclusion criteria – data collected for other purposes than developing and validating a prediction model (if a protocol is given this may reduce the ROB) - RCTs have narrower eligibility for participants, they also need to ensure treatment is included as a predictor, if not this is high ROB - ill-defined case-control/cohort studies are at high ROB - non-nested case–control design |
| **1.2 Were all inclusions and exclusions of participants appropriate?**  **NB** we are concerned with entry of participants into the study at this point, **NOT** into the analysis. We are not interested in loss to follow up at this point | **Decreases ROB (Y/PY)**   - inclusion/exclusion appropriate to get representative sample of target population - participants correspond to unselected participants of interest   **Increases ROB (N/PN)**   - inappropriate inclusion/exclusion of participants e.g., subgroups of populations that are not representative of the target population - includes participants who have already had the outcome e.g., including pre-operative transfusion patient when predicting intra- post-operative transfusion or if the blood transfusion outcome is a self-reported outcome measure |
| **Predictors** | |
| **2.1 Were predictors defined and assessed in a similar way for all participants?** | **Decreases ROB (Y/PY)**   - predictors defined and assessed in the same way - definitions of predictors and their assessment were similar for all participants   **Increases ROB (N/PN)**   - predictors not defined and assessed in the same way e.g., pre-op Hb measured using blood test or blood gas - predictors involving subjective judgement/assessment or skilled training, relying on the ability of the assessor - data from multiple sources likely to have used different definitions |
| **2.2 Were predictor assessments made without knowledge of outcome data?** | **Decreases ROB (Y/PY)**   - outcome information was stated as not used during predictor assessment or was clearly not (yet) available to those assessing predictors - blinding of the outcome   **Increases ROB (N/PN)**   - clear that outcome information was used when assessing predictors - lack of blinding of the outcome. Though for prognostic modelling, in a prospective cohort, blinding may occur naturally if the time between predictor assessment and outcome is sufficiently long enough – years – this is unlikely to be the case for us. - retrospectively recorded predictors   Studies often fail to report blinding = ‘No information’ |
| **2.3 Are all predictors available at the time the model is intended to be used?** | **Decreases ROB (Y/PY)**   - *For development studies,* the model can used in the real world and predictors are available in clinical settings (e.g., model uses pre-op predictors over intra-op predictors as predictors closer to the outcome are more strongly associated with it) - included predictors would be available at the time the model is intended to be used for prediction   **Increases ROB (N/PN)**   - *For validation studies,* predictor data needed for the model is missing from the validation dataset - predictors would not be available at the time the model is intended to be used for prediction |
| **Outcome** | |
| **3.1 Was the outcome determined appropriately?** | **Decreases ROB (Y/PY)**   - method of outcome determination has been used which is considered optimal or acceptable by guidelines or previous publications on the topic   **Increases ROB (N/PN)**   - a clearly suboptimal method has been used that causes unacceptable error in determining outcome status in participants - subjective outcomes e.g., imaging outcomes, outcomes at surgeon discretion, or ones which need special skill training |
| **3.2 Was a prespecified or standard outcome definition used?** | **Decreases ROB (Y/PY)**   - pre-specified/standard objective outcome - substantiated from clinical guidelines/previous studies/available protocol - prespecified categories are used to group outcomes   **Increases ROB (N/PN)**   - composite outcomes - uses atypical thresholds, or creates multiple thresholds on continuous outcome - consensus based outcomes e.g., surgical discretion |
| **3.3 Were predictors excluded from the outcome definition?**  **NB** check if pre -op Hb is a predictor in the model and check the outcome definition. | **Decreases ROB (Y/PY)**   - outcome determined without any predictor information - transfusion decision informed by post-op Hb test result, **NOT** pre-op Hb**.**   **Increases ROB (Y/PY)**   - ≥1 of the predictors forms part of the outcome definition - outcome determined using consensus panel e.g., consensus driven transfusion decision was made where the surgeon uses all available information to decide and preop Hb is in the model - outcome determination varies for each patient e.g., surgeon discretion |
| **3.4 Was the outcome defined and determined in a similar way for all participants?** | **Decreases ROB (Y/PY)**   - outcomes were defined and determined in a similar way for all participants   **Increases ROB (N/PN)**   - Data collected for non-research purposes e.g., routinely collected data from registries - More subjective outcomes |
| **3.5 Was the outcome determined without knowledge of predictor information?**  **NB** think of this like an RCT does not give knowledge of the treatment | **Decreases ROB (Y/PY)**   - predictor information was not known when determining the outcome status - outcome status determination is clearly reported as determined without knowledge of predictor information - outcome is a specific cause not requiring interpretation e.g., death   **Increases ROB (N/PN)**   - predictor information was used when determining the outcome status - outcomes were clearly defined and determined in a different way for some participants - outcomes requiring interpretation - predictor information would be available at time of outcome determination (consider the potential consequences) |
| **3.6 Was the time interval between predictor assessment and outcome determination appropriate?** | **Decreases ROB (Y/PY)**   - time interval between predictor assessment and outcome determination was appropriate to enable the correct type and representative number of relevant outcomes to be recorded   **Increases ROB (N/PN)**   - time between predictor assessment and outcome determination is too long/too short to enable the correct type and representative number of relevant outcomes – need clinical judgement - if the outcome is underrepresented = too short |
| **Analysis** | |
| **4.1 Were there a reasonable number of participants with the outcome?** | **Decreases ROB (Y/PY)**   - *For model development studies,* if the number of participants with the outcome relative to the number of candidate predictor parameters is ≥20 **(EPV ≥20)*** - *For model validation studies,* if the number of participants with the outcome is **≥100**   **Increases ROB (N/PN)**   - *For model development studies,* if the number of participants with the outcome relative to the number of candidate predictor parameters is **<10)*** - *For model validation studies,* if the number of participants with the outcome is **<100**   *For EPVs between 10 and 20, the item should be rated as either probably yes or probably no, depending on the outcome frequency, overall model performance, and distribution of the predictors in the model |
| **4.2 Were continuous and categorical handled appropriately?** | **Decreases ROB (Y/PY)**   - Continuous kept as continuous predictors - Continuous predictors examined for nonlinearity – look for ‘fractional polynomials’ or ‘restricted cubic splines’   **Increases ROB (N/PN)**   - Continuous predictors are dichotomised - Continuous predictors are categorised, especially using widely accepted clinical cut-offs**,** data driven cut-offs increase the ROB - *For validation studies,* predictors are collected using different format |
| **4.3 Were enrolled participants included in the analysis?** | **Decreases ROB (Y/PY)**   - all participants enrolled in the study are included in the data analysis, or a low number are excluded   **Increases ROB (N/PN)**   - some or a subgroup of participants are inappropriately excluded from the analysis, including participants with ‘unclear’ findings, missing data, or outliers |
| **4.4 Were participants with missing data handled appropriately?**  **NB** if missing data information not reported then assume complete case analysis was conducted | **Decreases ROB (Y/PY)**   - no missing values of predictors or outcomes and the study explicitly reports that participants are not excluded based on missing data - missing values are handled using multiple imputation - comparing results with and without missing data   **Increases ROB (N/PN)**   - missing data are omitted from the analysis - method of handling missing data is clearly flawed, e.g., missing indicator method or inappropriate use of last value carried forward - study had no explicit mention of methods to handle missing data |
| **4.5 Was selection of predictors based on univariable analysis avoided?**  **NB** this question applies for development studies only | **Decreases ROB (Y/PY)**   - predictors are not selected based on univariable analysis prior to multivariable modelling - predictors selected on existing knowledge, they are reliable, consistent, applicable, available, and credible - credible/a-priori predictors are forced into the model   **Increases ROB (N/PN)**   - predictors are selected based on univariable analysis prior to multivariable modelling |
| **4.6 Were complexities in the data (e.g., censoring, competing risks, sampling of control participants) accounted appropriately?** | **Decreases ROB (Y/PY)**   - complexities in the data are accounted for appropriately - clear that any potential data complexities have been identified appropriately as unimportant - Cox regression for time to event outcomes - Cox regression account for competing risks (e.g., hip replacement is often done in an older population who have a competing risk with death, that is that they might die before having the hip replacement) - Multilevel or random effects models for multiple outcome measures   **Increases ROB (N/PN)**   - complexities in the data that could affect model performance are ignored |
| **4.7 Were relevant model performance measures evaluated appropriately?** | **Decreases ROB (Y/PY)**   - both calibration and discrimination are evaluated appropriately (including relevant measures tailored for models predicting survival outcomes)   **Increases ROB (N/PN)**   - both calibration and discrimination are not evaluated - only goodness-of-fit tests, such as the Hosmer–Lemeshow test, are used to evaluate calibration - models predicting survival outcomes performance measures accounting for censoring are not used - if classification measures (like sensitivity, specificity, or predictive values) were presented using predicted probability thresholds derived from the data set at hand/non-clinical cut-offs |
| **4.8 Was model overfitting, underfitting, and optimism in model performance accounted for?**  **NB** this question applies for development studies only | **Decreases ROB (Y/PY)**   - internal validation techniques, such as bootstrapping and cross-validation including all model development procedures, have been used to account for any optimism in model fitting, and subsequent adjustment of the model performance estimates have been applied   **Increases ROB (N/PN)**   - no internal validation has been performed, or if internal validation consists only of a single random split-sample of participant data - bootstrapping or cross-validation did not include all model development procedures including any variable selection |
| **4.9 Do predictors and their assigned weights in the final model correspond to the results from the reported multivariable analysis?** | **Decreases ROB (Y/PY)**   - predictors and regression coefficients in the final model correspond to reported results from multivariable analysis   **Increases ROB (N/PN)**   - predictors and regression coefficients in the final model do not correspond to reported results from multivariable analysis |

**Supplementary table 5. List of all studies and their study designs**

| **Title** | **Study Design** |
| --- | --- |
| Accuracy Enhanced Lung Cancer Prognosis for Improving Patient Survivability Using Proposed Gaussian Classifier System [1] | Development only study |
| Analysis of survival for lung cancer resections cases with fuzzy and soft set theory in surgical decision making [2] | Development only study |
| Application of machine learning techniques to analyze anastomosis integrity after Total gastrectomy for prediction of clinical leakage [3] | Development only study |
| Artificial neural network models to predict nodal status in clinically node-negative breast cancer [4] | Development only study |
| askMUSIC: Leveraging a Clinical Registry to Develop a New Machine Learning Model to Inform Patients of Prostate Cancer Treatments Chosen by Similar Men [5] | Development only study |
| Assessment of Deep Learning Using Nonimaging Information and Sequential Medical Records to Develop a Prediction Model for Nonmelanoma Skin Cancer [6] | Development only study |
| Automated data extraction and ensemble methods for predictive modeling of breast cancer outcomes after radiation therapy [7] | Development only study |
| Can machine learning predict resecability of a peritoneal carcinomatosis? [8] | Development only study |
| Characteristics and long-term outcomes of advanced pleural mesothelioma in Latin America (MeSO-CLICaP) [9] | Development only study |
| Clinical characteristics and disease specific prognostic nomogram for primary gliosarcoma: a SEER population-based analysis [10] | Development only study |
| Creating Prognostic Systems for Well-Differentiated Thyroid Cancer Using Machine Learning [11] | Development only study |
| Deep learning-based survival prediction of oral cancer patients [12] | Development only study |
| Deep stacked sparse auto-encoders for prediction of postoperative survival expectancy in thoracic lung cancer surgery [13] | Development only study |
| Developing case-finding algorithms for second events of oropharyngeal cancer using administrative data: A population-based validation study [14] | Development only study |
| Development and Assessment of a Machine Learning Model to Help Predict Survival Among Patients With Oral Squamous Cell Carcinoma [15] | Development only study |
| Development and validation of case-finding algorithms for recurrence of breast cancer using routinely collected administrative data [16] | Development only study |
| Development of a Novel Prognostic Risk Score for Predicting Complications of Penectomy in the Surgical Management of Penile Cancer [17] | Development only study |
| Development of Deep Learning Algorithm for Detection of Colorectal Cancer in EHR Data [18] | Development only study |
| Development of Machine Learning Algorithms for Prediction of 30-Day Mortality After Surgery for Spinal Metastasis [19] | Development only study |
| Extent of Resection in Meningioma: Predictive Factors and Clinical Implications [20] | Development only study |
| Gait speed and survival of older surgical patient with cancer: Prediction after machine learning [21] | Development only study |
| Machine Learning Algorithm Identifies Patients at High Risk for Early Complications After Intracranial Tumor Surgery: Registry-Based Cohort Study [22] | Development only study |
| Machine learning methods applied to audit of surgical outcomes after treatment for cancer of the head and neck [23] | Development only study |
| Machine Learning to Predict Delays in Adjuvant Radiation following Surgery for Head and Neck Cancer [24] | Development only study |
| A machine-learning based prediction model of fistula formation after interstitial brachytherapy for locally advanced gynecological malignancies [25] | Development only study |
| A novel prediction method for lymph node involvement in endometrial cancer: machine learning [26] | Development only study |
| Posterior fossa meningiomas: perioperative predictors of extent of resection, overall survival and progression-free survival [27] | Development only study |
| Predicting 90-Day and 1-Year Mortality in Spinal Metastatic Disease: Development and Internal Validation [28] | Development only study |
| Predicting breast cancer metastasis by using serum biomarkers and clinicopathological data with machine learning technologies [29] | Development only study |
| Predictors of the therapeutic effect of corticosteroids on radiation-induced optic neuropathy following nasopharyngeal carcinoma [30] | Development only study |
| Predictive model algorithms identifying early and advanced stage ER+/HER2- breast cancer in claims data [31] | Development only study |
| Predicting cervical cancer screening among sexual minority women using Classification and Regression Tree analysis [32] | Development only study |
| Predicting Disease-Free Lung Cancer Survival Using Patient Reported Outcome (PRO) Measurements with Comparisons of Five Machine Learning Techniques (MLT) [33] | Development only study |
| Prediction of 10-year Overall Survival in Patients with Operable Cervical Cancer using a Probabilistic Neural Network [34] | Development only study |
| Predicting radiation pneumonitis in locally advanced stage II-III non-small cell lung cancer using machine learning [35] | Development only study |
| Predicting Survival of Patients with Spinal Ependymoma Using Machine Learning Algorithms with the SEER Database [36] | Development only study |
| Prediction of future gastric cancer risk using a machine learning algorithm and comprehensive medical check-up data: A case-control study [37] | Development only study |
| Prediction of irinotecan toxicity in metastatic colorectal cancer patients based on machine learning models with pharmacokinetic parameters [38] | Development only study |
| A Predictive Model for Postembolization Syndrome after Transarterial Hepatic Chemoembolization of Hepatocellular Carcinoma [39] | Development only study |
| A prospective study examining cachexia predictors in patients with incurable cancer [40] | Development only study |
| Using Machine Learning to Predict Progression in the Gastric Precancerous Process in a Population from a Developing Country Who Underwent a Gastroscopy for Dyspeptic Symptoms [41] | Development only study |
| Recursive Partitioning Analysis (RPA) of Prognostic Factors for Overall Survival in Patients with Spinal Metastasis: A New System for Stratified Treatment [42] | Development only study |
| Response to repeat echoendoscopic celiac plexus neurolysis in pancreatic cancer patients: A machine learning approach [43] | Development only study |
| Risk Factors for Local Relapse and Inferior Disease-free Survival After Breast-conserving Management of Breast Cancer: Recursive Partitioning Analysis of 2161 Patients [44] | Development only study |
| Survivability prediction of colon cancer patients using neural networks [45] | Development only study |
| Survival outcome prediction in cervical cancer: Cox models vs deep-learning model [46] | Development only study |
| Use of Machine Learning for Prediction of Patient Risk of Postoperative Complications After Liver, Pancreatic, and Colorectal Surgery [47] | Development only study |
| Use of machine learning to predict early biochemical recurrence after robot-assisted prostatectomy [48] | Development only study |
| Age and Lymphovascular Invasion Accurately Predict Sentinel Lymph Node Metastasis in T2 Melanoma Patients [49] | Development and  validation study |
| Early Warning Models to Estimate the 30-Day Mortality Risk After Stent Placement for Patients with Malignant Biliary Obstruction [50] | Development and  validation study |
| Machine learning application for prediction of locoregional recurrences in early oral tongue cancer: a Web-based prognostic tool [51] | Development and  validation study |
| Nomograms for predicting the overall and cause-specific survival in patients with malignant peripheral nerve sheath tumor: a population-based study [52] | Development and  validation study |
| Patient-based prediction algorithm of relapse after allo-HSCT for acute Leukemia and its usefulness in the decision-making process using a machine learning approach [53] | Development and  validation study |
| A predictive model of overall survival in patients with metastatic castration-resistant prostate cancer [54] | Development and  validation study |
| Predicting Inpatient Length of Stay After Brain Tumor Surgery: Developing Machine Learning Ensembles to Improve Predictive Performance [55] | Development and  validation study |
| Predicting Overall Survival in Patients with Metastatic Rectal Cancer: a Machine Learning Approach [56] | Development and  validation study |
| Prediction of survival outcomes in patients with epithelial ovarian cancer using machine learning methods [57] | Development and  validation study |
| Prediction of the 1-Year Risk of Incident Lung Cancer: Prospective Study Using Electronic Health Records from the State of Maine [58] | Development and  validation study |
| A Proposal to Reflect Survival Difference and Modify the Staging System for Lung Adenocarcinoma and Squamous Cell Carcinoma: Based on the Machine Learning [59] | Development and  validation study |
| Scoring colorectal cancer risk with an artificial neural network based on self-reportable personal health data [60] | Development and  validation study |
| Semi-supervised learning to improve generalizability of risk prediction models [61] | Development and  validation study |
| Use of Machine-Learning Algorithms in Intensified Preoperative Therapy of Pancreatic Cancer to Predict Individual Risk of Relapse [62] | Development and  validation study |

**Supplementary table 6.** Model characteristics developed in the 62 included publications.

| **Model characteristics** | **All (n=152 models)** | **Development only (n=115)** | **Development and external validation (n=37)** |
| --- | --- | --- | --- |
|  | **n (%)** | **n (%)** | **n (%)** |
| **Regression-based machine learning models** | **42 (27.6)** | **30 (26.1)** | **12 (32.4)** |
| Logistic regression | 27 | 19 | 8 |
| Cox regression | 8 | 5 | 3 |
| Linear regression | 3 | 3 | - |
| LASSO | 3 | 2 | 1 |
| Other* | 1 | 1 | - |
| **Flexible machine learning models** | **71 (46.7)** | **57 (49.6)** | **14 (37.8)** |
| Neural network (including deep learning) | 18 | 14 | 4 |
| Classification tree (e.g., CART, decision tree) | 28 | 25 | 3 |
| Support vector machine | 12 | 9 | 3 |
| Naive Bayes | 6 | 5 | 1 |
| K nearest neighbours | 3 | 1 | 2 |
| Other** | 4 | 3 | 1 |
| **Ensemble models** | **39 (25.7)** | **28 (24.4)** | **11 (29.7)** |
| Random forest (including random survival forest) | 23 | 19 | 4 |
| Gradient boosting machine | 8 | 3 | 5 |
| RUSBoost - boosted random forests | 1 | 1 | - |
| Bagging with J48 selected by Auto-WEKA | 1 | 1 | - |
| CoxBoost - boosted Cox regression | 1 | 1 | - |
| XGBoost: exTreme Gradient Boosting | 1 | - | 1 |
| Gradient boosting machine and Nystroem, combined using elastic net | 1 | - | 1 |
| Adaboost | 1 | 1 | - |
| Bagging, method not specified | 1 | 1 | - |
| Partitioning Around Medoid algorithm and complete linkage method | 1 | 1 | - |
| **Median number of models developed per study [IQR], range** | 2 [1-4], 1-6 | 2 [1-4], 1-6 | 2 [1-5], 1-6 |

CART - Classification and regression tree; LASSO - Least Absolute Shrinkage and Selection Operator

* other is best subset regression with leave-out cross-validation

** other includes voted perceptron; fuzzy logic, soft set theory and soft set computing; hierarchical clustering model based on the unsupervised learning for survival data using the distance matrix of survival curves; Bayes point machine

**Supplementary table 7.** Risk of bias assessment by machine learning model type.

|  | **Regression-based models (n=42)** | | | **Flexible models (n=71)** | | | **Ensemble models (n=39)** | | |
| --- | --- | --- | --- | --- | --- | --- | --- | --- | --- |
|  | **Y/PY** | **N/PN** | **NI** | **Y/PY** | **N/PN** | **NI** | **Y/PY** | **N/PN** | **NI** |
|  | **%, 95% CI** | **%, 95% CI** | **%, 95% CI** | **%, 95% CI** | **%, 95% CI** | **%, 95% CI** | **%, 95% CI** | **%, 95% CI** | **%, 95% CI** |
| **1.       PARTICIPANTS** |  |  |  |  |  |  |  |  |  |
| 1.1.  Were appropriate data sources used, e.g., cohort, randomized controlled trial, or nested case–control study data? | 83 (68 to 92) | 10 (4 to 23) | 7 (2 to 21) | 70 (59 to 80) | 13 (7 to 23) | 17 (10 to 28) | 77 (61 to 88) | 15 (7 to 31) | 8 (2 to 22) |
| 1.2.  Were all inclusions and exclusions of participants appropriate? | 74 (58 to 85) | 5 (1 to 18) | 21 (11 to 37) | 61 (49 to 71) | 13 (7 to 23) | 27 (18 to 38) | 67 (50 to 80) | 5 (1 to 19) | 28 (16 to 45) |
| **2.       PREDICTORS** |  |  |  |  |  |  |  |  |  |
| 2.1.  Were predictors defined and assessed in a similar way for all participants? | 81 (66 to 90) | 5 (1 to 18) | 14 (6 to 29) | 77 (66 to 86) | 13 (7 to 23) | 10 (5 to 19) | 72 (55 to 84) | 8 (2 to 22) | 21 (10 to 37) |
| 2.2. Were predictor assessments made without knowledge of outcome data? | 50 (35 to 65) | 0 (0 to 0) | 50 (35 to 65) | 44 (32 to 56) | 1 (0 to 10) | 55 (43 to 66) | 54 (38 to 69) | 0 (0 to 0) | 46 (31 to 62) |
| 2.3.  Are all predictors available at the time the model is intended to be used? | 57 (42 to 71) | 0 (0 to 0) | 43 (29 to 58) | 59 (47 to 70) | 0 (0 to 0) | 41 (30 to 53) | 64 (48 to 78) | 0 (0 to 0) | 36 (22 to 52) |
| **3.       OUTCOMES** |  |  |  |  |  |  |  |  |  |
| 3.1.  Was the outcome determined appropriately? | 90 (77 to 96) | 0 (0 to 0) | 10 (4 to 23) | 83 (72 to 90) | 3 (1 to 11) | 14 (8 to 24) | 85 (69 to 93) | 5 (1 to 19) | 10 (4 to 25) |
| 3.2. Was a prespecified or standard outcome definition used? | 76 (61 to 87) | 10 (4 to 23) | 14 (6 to 29) | 83 (72 to 90) | 7 (3 to 16) | 10 (5 to 19) | 79 (63 to 90) | 10 (4 to 25) | 10 (4 to 25) |
| 3.3.  Were predictors excluded from the outcome definition? | 81 (66 to 90) | 5 (1 to 18) | 14 (6 to 29) | 73 (62 to 82) | 4 (1 to 13) | 23 (14 to 34) | 79 (63 to 90) | 3 (0 to 17) | 18 (9 to 34) |
| 3.4.  Was the outcome defined and determined in a similar way for all participants? | 83 (68 to 92) | 5 (1 to 18) | 12 (5 to 26) | 72 (60 to 81) | 8 (4 to 18) | 20 (12 to 31) | 74 (58 to 86) | 8 (2 to 22) | 18 (9 to 34) |
| 3.5.  Was the outcome determined without knowledge of predictor information? | 74 (58 to 85) | 5 (1 to 18) | 21 (11 to 37) | 68 (56 to 78) | 4 (1 to 13) | 28 (19 to 40) | 69 (53 to 82) | 3 (0 to 17) | 28 (16 to 45) |
| 3.6.  Was the time interval between predictor assessment and outcome determination appropriate? | 69 (53 to 81) | 5 (1 to 18) | 26 (15 to 42) | 62 (50 to 73) | 0 (0 to 0) | 38 (27 to 50) | 69 (53 to 82) | 8 (2 to 22) | 23 (12 to 39) |
| **4.       ANALYSIS** |  |  |  |  |  |  |  |  |  |
| 4.1.  Were there a reasonable number of participants with the outcome? | 31 (19 to 47) | 52 (37 to 67) | 17 (8 to 32) | 34 (24 to 46) | 54 (42 to 65) | 13 (7 to 23) | 18 (9 to 34) | 44 (29 to 60) | 38 (24 to 55) |
| 4.2.  Were continuous and categorical predictors handled appropriately? | 21 (11 to 37) | 33 (21 to 49) | 45 (31 to 61) | 15 (9 to 26) | 41 (30 to 53) | 44 (32 to 56) | 26 (14 to 42) | 36 (22 to 52) | 38 (24 to 55) |
| 4.3.  Were all enrolled participants included in the analysis? | 31 (19 to 47) | 33 (21 to 49) | 36 (22 to 52) | 24 (15 to 35) | 32 (22 to 44) | 44 (32 to 56) | 33 (20 to 50) | 31 (18 to 47) | 36 (22 to 52) |
| 4.4.  Were participants with missing data handled appropriately? | 19 (10 to 34) | 52 (37 to 67) | 29 (17 to 44) | 11 (6 to 21) | 41 (30 to 53) | 48 (36 to 60) | 21 (10 to 37) | 49 (33 to 65) | 31 (18 to 47) |
| 4.5.  Was selection of predictors based on univariable analysis avoided? | 48 (33 to 63) | 38 (24 to 54) | 14 (6 to 29) | 44 (32 to 56) | 28 (19 to 40) | 28 (19 to 40) | 44 (29 to 60) | 33 (20 to 50) | 23 (12 to 39) |
| 4.6.  Were complexities in the data (e.g., censoring, competing risks, sampling of control participants) accounted for appropriately? | 14 (6 to 29) | 17 (8 to 32) | 69 (53 to 81) | 1 (0 to 10) | 18 (11 to 29) | 80 (69 to 88) | 8 (2 to 22) | 21 (10 to 37) | 72 (55 to 84) |
| 4.7.  Were relevant model performance measures evaluated appropriately? | 19 (10 to 34) | 52 (37 to 67) | 29 (17 to 44) | 20 (12 to 31) | 61 (49 to 71) | 20 (12 to 31) | 15 (7 to 31) | 56 (40 to 71) | 28 (16 to 45) |
| 4.8.  Were model overfitting and optimism in model performance accounted for? | 52 (37 to 67) | 38 (24 to 54) | 10 (4 to 23) | 27 (18 to 38) | 61 (49 to 71) | 13 (7 to 23) | 28 (16 to 45) | 64 (48 to 78) | 8 (2 to 22) |
| 4.9.  Do predictors and their assigned weights in the final model correspond to the results from the reported multivariable analysis? | 17 (8 to 32) | 10 (4 to 23) | 74 (58 to 85) | 23 (14 to 34) | 3 (1 to 11) | 75 (63 to 84) | 3 (0 to 17) | 5 (1 to 19) | 92 (78 to 98) |
| **Domain** | **Low** | **High** | **NI** | **Low** | **High** | **NI** | **Low** | **High** | **NI** |
|  | **%, 95% CI** | **%, 95% CI** | **%, 95% CI** | **%, 95% CI** | **%, 95% CI** | **%, 95% CI** | **%, 95% CI** | **%, 95% CI** | **%, 95% CI** |
| Participants | 69 (53 to 81) | 7 (2 to 21) | 24 (13 to 39) | 55 (43 to 66) | 15 (9 to 26) | 30 (20 to 41) | 64 (48 to 78) | 10 (4 to 25) | 26 (14 to 42) |
| Predictors | 55 (39 to 69) | 0 (0 to 0) | 45 (31 to 61) | 54 (42 to 65) | 1 (0 to 10) | 45 (34 to 57) | 51 (35 to 67) | 0 (0 to 0) | 49 (33 to 65) |
| Outcomes | 81 (66 to 90) | 5 (1 to 18) | 14 (6 to 29) | 70 (59 to 80) | 7 (3 to 16) | 23 (14 to 34) | 69 (53 to 82) | 13 (5 to 28) | 18 (9 to 34) |
| Analysis | 10 (4 to 23) | 79 (63 to 89) | 12 (5 to 26) | 8 (4 to 18) | 85 (74 to 91) | 7 (3 to 16) | 10 (4 to 25) | 77 (61 to 88) | 13 (5 to 28) |
| Overall | 5 (1 to 18) | 83 (68 to 92) | 12 (5 to 26) | 4 (1 to 13) | 85 (74 to 91) | 11 (6 to 21) | 5 (1 to 19) | 85 (69 to 93) | 10 (4 to 25) |

**References for supplementary information**

1 Kaviarasi R, Gandhi RR . Accuracy Enhanced Lung Cancer Prognosis for Improving Patient Survivability Using Proposed Gaussian Classifier System. J Med Syst 2019;43:201. <https://doi.org/10.1007/s10916-019-1297-2>

2 Alcantud JCR, Varela G, Santos-Buitrago B, et al. Analysis of survival for lung cancer resections cases with fuzzy and soft set theory in surgical decision making. PLoS One 2019;14:e0218283. <https://doi.org/10.1371/journal.pone.0218283>

3 Celik S, Sohail A, Ashraf S, et al. Application of machine learning techniques to analyze anastomosis integrity after Total gastrectomy for prediction of clinical leakage. Health Technol 2019;9:757–63. <https://doi.org/10.1007/s12553-019-00334-3>

4 Dihge L, Ohlsson M, Edén P, et al. Artificial neural network models to predict nodal status in clinically node-negative breast cancer. BMC Cancer 2019;19:610. <https://doi.org/10.1186/s12885-019-5827-6>

5 Auffenberg GB, Ghani KR, Ramani S, et al. askMUSIC: Leveraging a Clinical Registry to Develop a New Machine Learning Model to Inform Patients of Prostate Cancer Treatments Chosen by Similar Men. Eur Urol 2019;75:901–7. <https://doi.org/10.1016/j.eururo.2018.09.050>

6 Wang H-H, Wang Y-H, Liang C-W, et al. Assessment of Deep Learning Using Nonimaging Information and Sequential Medical Records to Develop a Prediction Model for Nonmelanoma Skin Cancer. JAMA Dermatol 2019. 155(11):1277-1283. <https://doi.org/10.1001/jamadermatol.2019.2335>

7 Lindsay WD, Ahern CA, Tobias JS, et al. Automated data extraction and ensemble methods for predictive modeling of breast cancer outcomes after radiation therapy. Med Phys 2019;46:1054–63. <https://doi.org/10.1002/mp.13314>

8 Maubert A, Birtwisle L, Bernard JL, et al. Can machine learning predict resecability of a peritoneal carcinomatosis? Surg Oncol 2019;29:120–5. <https://doi.org/10.1016/j.suronc.2019.04.008>

9 Rojas L, Cardona AF, Trejo-Rosales R, et al. Characteristics and long-term outcomes of advanced pleural mesothelioma in Latin America (MeSO-CLICaP). Thorac Cancer 2019;10:508–18. <https://doi.org/10.1111/1759-7714.12967>

10 Feng S-S, Li H, Fan F, et al. Clinical characteristics and disease-specific prognostic nomogram for primary gliosarcoma: a SEER population-based analysis. Sci Rep 2019;9:10744. <https://doi.org/10.1038/s41598-019-47211-7>

11 Yang CQ, Gardiner L, Wang H, et al. Creating Prognostic Systems for Well-Differentiated Thyroid Cancer Using Machine Learning. Front Endocrinol (Lausanne) 2019;10:288. <https://doi.org/10.3389/fendo.2019.00288>

12 Kim DW, Lee S, Kwon S, et al. Deep learning-based survival prediction of oral cancer patients. Sci Rep 2019;9:6994. <https://doi.org/10.1038/s41598-019-43372-7>

13 Iraji MS. Deep stacked sparse auto-encoders for prediction of post-operative survival expectancy in thoracic lung cancer surgery. J Appl Biomed 2019;17:75–75. <https://doi.org/10.32725/jab.2018.007>

14 Xu Y, Kong S, Cheung WY, et al. Developing case-finding algorithms for second events of oropharyngeal cancer using administrative data: A population-based validation study. Head Neck 2019;41:2291–8. <https://doi.org/10.1002/hed.25682>

15 Karadaghy OA, Shew M, New J, et al. Development and Assessment of a Machine Learning Model to Help Predict Survival Among Patients With Oral Squamous Cell Carcinoma. JAMA Otolaryngol Head Neck Surg 2019;145(12):1115-1120. <https://doi.org/10.1001/jamaoto.2019.0981>

16 Xu Y, Kong S, Cheung WY, et al. Development and validation of case-finding algorithms for recurrence of breast cancer using routinely collected administrative data. BMC Cancer 2019;19:210. <https://doi.org/10.1186/s12885-019-5432-8>

17 Velazquez N, Press B, Renson A, et al. Development of a Novel Prognostic Risk Score for Predicting Complications of Penectomy in the Surgical Management of Penile Cancer. Clin Genitourin Cancer 2019;17:e123–9. <https://doi.org/10.1016/j.clgc.2018.09.018>

18 Wang Y-H, Nguyen P-A, Islam MM, et al. Development of Deep Learning Algorithm for Detection of Colorectal Cancer in EHR Data. Stud Health Technol Inform 2019;264:438–41. <https://doi.org/10.3233/SHTI190259>

19 Karhade AV, Thio QCBS, Ogink PT, et al. Development of Machine Learning Algorithms for Prediction of 30-Day Mortality After Surgery for Spinal Metastasis. Neurosurgery 2019;85:E83–91. <https://doi.org/10.1093/neuros/nyy469>

20 Lemée J-M, Corniola MV, Da Broi M, et al. Extent of Resection in Meningioma: Predictive Factors and Clinical Implications. Sci Rep 2019;9:5944. <https://doi.org/10.1038/s41598-019-42451-z>

21 Sasani K, Catanese HN, Ghods A, et al. Gait speed and survival of older surgical patient with cancer: Prediction after machine learning. J Geriatr Oncol 2019;10:120–5. <https://doi.org/10.1016/j.jgo.2018.06.012>

22 van Niftrik CHB, van der Wouden F, Staartjes VE, et al. Machine Learning Algorithm Identifies Patients at High Risk for Early Complications After Intracranial Tumor Surgery: Registry-Based Cohort Study. Neurosurgery 2019;85:E756–64. <https://doi.org/10.1093/neuros/nyz145>

23 Tighe D, Lewis-Morris T, Freitas A. Machine learning methods applied to audit of surgical outcomes after treatment for cancer of the head and neck. Br J Oral and Maxillofac Surg 2019;57:771–7. <https://doi.org/10.1016/j.bjoms.2019.05.026>

24 Shew M, New J, Bur AM. Machine Learning to Predict Delays in Adjuvant Radiation following Surgery for Head and Neck Cancer. Otolaryngol Head Neck Surg 2019;160:1058–64. <https://doi.org/10.1177/0194599818823200>

25 Tian Z, Yen A, Zhou Z, et al. A machine-learning–based prediction model of fistula formation after interstitial brachytherapy for locally advanced gynecological malignancies. Brachytherapy 2019;18:530–8. <https://doi.org/10.1016/j.brachy.2019.04.004>

26 Günakan E, Atan S, Haberal AN, et al. A novel prediction method for lymph node involvement in endometrial cancer: machine learning. Int J Gynecol Cancer 2019;29. <https://doi.org/10.1136/ijgc-2018-000033>

27 Corniola MV, Lemée J-M, Da Broi M, et al. Posterior fossa meningiomas: perioperative predictors of extent of resection, overall survival and progression-free survival. Acta Neurochir (Wien) 2019;161:1003–11. <https://doi.org/10.1007/s00701-019-03862-z>

28 Karhade AV, Thio QCBS, Ogink PT, et al. Predicting 90-Day and 1-Year Mortality in Spinal Metastatic Disease: Development and Internal Validation. Neurosurgery 2019;85:E671–81. <https://doi.org/10.1093/neuros/nyz070>

29 Tseng Y-J, Huang C-E, Wen C-N, et al. Predicting breast cancer metastasis by using serum biomarkers and clinicopathological data with machine learning technologies. Int J Med Inform 2019;128:79–86. <https://doi.org/10.1016/j.ijmedinf.2019.05.003>

30 Zheng B, Lin J, Li Y, et al. Predictors of the therapeutic effect of corticosteroids on radiation-induced optic neuropathy following nasopharyngeal carcinoma. Support Care Cancer 2019;27:4213–9. <https://doi.org/10.1007/s00520-019-04699-z>

31 Beachler DC, de Luise C, Yin R, et al. Predictive model algorithms identifying early and advanced stage ER+/HER2- breast cancer in claims data. Pharmacoepidemiol Drug Saf 2019;28:171–8. <https://doi.org/10.1002/pds.4681>

32 Greene MZ, Hughes TL, Hanlon A, et al. Predicting cervical cancer screening among sexual minority women using Classification and Regression Tree analysis. Prev Med Rep 2019;13:153–9. <https://doi.org/10.1016/j.pmedr.2018.11.007>

33 Sim J-A, Yun YH. Predicting Disease-Free Lung Cancer Survival Using Patient Reported Outcome (PRO) Measurements with Comparisons of Five Machine Learning Techniques (MLT). Stud Health Technol Inform 2019;264:1588–9. <https://doi.org/10.3233/SHTI190548>

34 Obrzut B, Kusy M, Semczuk A, et al. Prediction of 10-year Overall Survival in Patients with Operable Cervical Cancer using a Probabilistic Neural Network. J Cancer 2019;10:4189–95. <https://doi.org/10.7150/jca.33945>

35 Luna JM, Chao H-H, Diffenderfer ES, et al. Predicting radiation pneumonitis in locally advanced stage II-III non-small cell lung cancer using machine learning. Radiother Oncol 2019;133:106–12. <https://doi.org/10.1016/j.radonc.2019.01.003>

36 Ryu SM, Lee S-H, Kim E-S, et al. Predicting Survival of Patients with Spinal Ependymoma Using Machine Learning Algorithms with the SEER Database. World Neurosurg 2018; S1878-8750(18)32914-0. <https://doi.org/10.1016/j.wneu.2018.12.091>

37 Taninaga J, Nishiyama Y, Fujibayashi K, et al. Prediction of future gastric cancer risk using a machine learning algorithm and comprehensive medical check-up data: A case-control study. Sci Rep 2019;9:12384. <https://doi.org/10.1038/s41598-019-48769-y>

38 Oyaga-Iriarte E, Insausti A, Sayar O, et al. Prediction of irinotecan toxicity in metastatic colorectal cancer patients based on machine learning models with pharmacokinetic parameters. J Pharmacol Sci 2019;140:20–5. <https://doi.org/10.1016/j.jphs.2019.03.004>

39 Khalaf MH, Sundaram V, AbdelRazek Mohammed MA, et al. A Predictive Model for Postembolization Syndrome after Transarterial Hepatic Chemoembolization of Hepatocellular Carcinoma. Radiology 2019;290:254–61. <https://doi.org/10.1148/radiol.2018180257>

40 Vagnildhaug OM, Brunelli C, Hjermstad MJ, et al. A prospective study examining cachexia predictors in patients with incurable cancer. BMC Palliat Care 2019;18:46. <https://doi.org/10.1186/s12904-019-0429-2>

41 Thapa S, Fischback LA, Delongchamp R, et al. Using Machine Learning to Predict Progression in the Gastric Precancerous Process in a Population from a Developing Country Who Underwent a Gastroscopy for Dyspeptic Symptoms. Gastroenterol Res Pract 2019:8321942. <https://doi.org/10.1155/2019/8321942>

42 Yang X-G, Wang F, Feng J-T, et al. Recursive Partitioning Analysis (RPA) of Prognostic Factors for Overall Survival in Patients with Spinal Metastasis: A New System for Stratified Treatment. World Neurosurg 2019;127:e124–31. <https://doi.org/10.1016/j.wneu.2019.02.183>

43 Facciorusso A, Del Prete V, Antonino M, et al. Response to repeat echoendoscopic celiac plexus neurolysis in pancreatic cancer patients: A machine learning approach. Pancreatology 2019;19:866–72. <https://doi.org/10.1016/j.pan.2019.07.038>

44 Hammer J, Geinitz H, Nieder C, et al. Risk Factors for Local Relapse and Inferior Disease-free Survival After Breast-conserving Management of Breast Cancer: Recursive Partitioning Analysis of 2161 Patients. Clin Breast Cancer 2019;19:58–62. <https://doi.org/10.1016/j.clbc.2018.08.001>

45 Al-Bahrani R, Agrawal A, Choudhary A. Survivability prediction of colon cancer patients using neural networks. Health Informatics J 2019;25:878–91. <https://doi.org/10.1177/1460458217720395>

46 Matsuo K, Purushotham S, Jiang B, et al. Survival outcome prediction in cervical cancer: Cox models vs deep-learning model. Am J Obstet Gynecol 2019;220:381.e1-381.e14. <https://doi.org/10.1016/j.ajog.2018.12.030>

47 Merath K, Hyer JM, Mehta R, et al. Use of Machine Learning for Prediction of Patient Risk of Postoperative Complications After Liver, Pancreatic, and Colorectal Surgery. J Gastrointest Surg 2020;24:1843–51. <https://doi.org/10.1007/s11605-019-04338-2>

48 Wong NC, Lam C, Patterson L, et al. Use of machine learning to predict early biochemical recurrence after robot-assisted prostatectomy. BJU Int 2019;123:51–7. <https://doi.org/10.1111/bju.14477>

49 Egger ME, Stevenson M, Bhutiani N, et al. Age and Lymphovascular Invasion Accurately Predict Sentinel Lymph Node Metastasis in T2 Melanoma Patients. Ann Surg Oncol 2019;26:3955–61. <https://doi.org/10.1245/s10434-019-07690-4>

50 Zhou H-F, Lu J, Zhu H-D, et al. Early Warning Models to Estimate the 30-Day Mortality Risk After Stent Placement for Patients with Malignant Biliary Obstruction. Cardiovasc Intervent Radiol 2019;42:1751–9. <https://doi.org/10.1007/s00270-019-02331-5>

51 Alabi RO, Elmusrati M, Sawazaki-Calone I, et al. Machine learning application for prediction of locoregional recurrences in early oral tongue cancer: a Web-based prognostic tool. Virchows Arch 2019;475:489–97. <https://doi.org/10.1007/s00428-019-02642-5>

52 Yan P, Huang R, Hu P, et al. Nomograms for predicting the overall and cause-specific survival in patients with malignant peripheral nerve sheath tumor: a population-based study. J Neurooncol 2019;143:495–503. <https://doi.org/10.1007/s11060-019-03181-4>

53 Fuse K, Uemura S, Tamura S, et al. Patient-based prediction algorithm of relapse after allo-HSCT for acute Leukemia and its usefulness in the decision-making process using a machine learning approach. Cancer Med 2019;8:5058–67. <https://doi.org/10.1002/cam4.2401>

54 Mahmoudian M, Seyednasrollah F, Koivu L, et al. A predictive model of overall survival in patients with metastatic castration-resistant prostate cancer [version 2; peer review: 2 approved]. F1000Res 2019;5:2674. <https://doi.org/10.12688/f1000research.8192.2>

55 Muhlestein WE, Akagi DS, Davies JM, et al. Predicting Inpatient Length of Stay After Brain Tumor Surgery: Developing Machine Learning Ensembles to Improve Predictive Performance. Neurosurgery 2019;85:384–93. <https://doi.org/10.1093/neuros/nyy343>

56 Zhao B, Gabriel RA, Vaida F, et al. Predicting Overall Survival in Patients with Metastatic Rectal Cancer: a Machine Learning Approach. J Gastrointest Surg 2019;24:1165–72. <https://doi.org/10.1007/s11605-019-04373-z>

57 Paik ES, Lee JW, Park JY, et al. Prediction of survival outcomes in patients with epithelial ovarian cancer using machine learning methods. J Gynecol Oncol 2019;30:e65. <https://doi.org/10.3802/jgo.2019.30.e65>

58 Wang X, Zhang Y, Hao S, et al. Prediction of the 1-Year Risk of Incident Lung Cancer: Prospective Study Using Electronic Health Records from the State of Maine. J Med Internet Res 2019;21:e13260. <https://doi.org/10.2196/13260>

59 Li M, Zhan C, Sui X, et al. A Proposal to Reflect Survival Difference and Modify the Staging System for Lung Adenocarcinoma and Squamous Cell Carcinoma: Based on the Machine Learning. Front Oncol 2019;9. <https://doi.org/10.3389/fonc.2019.00771>

60 Nartowt BJ, Hart GR, Roffman DA, et al. Scoring colorectal cancer risk with an artificial neural network based on self-reportable personal health data. PLoS One 2019;14:e0221421. <https://doi.org/10.1371/journal.pone.0221421>

61 Chi S, Li X, Tian Y, et al. Semi-supervised learning to improve generalizability of risk prediction models. J Biomed Inform 2019;92:103117. <https://doi.org/10.1016/j.jbi.2019.103117>

62 Sala Elarre P, Oyaga-Iriarte E, Yu KH, et al. Use of Machine-Learning Algorithms in Intensified Preoperative Therapy of Pancreatic Cancer to Predict Individual Risk of Relapse. Cancers (Basel) 2019;11(5):606. <https://doi.org/10.3390/cancers11050606>
